# Sex shapes CD64 expression and vaccine-induced monocytic responses

**DOI:** 10.1186/s13293-026-00897-7

**Published:** 2026-04-05

**Authors:** Charlotte Sophie Hansen, Julie Sellau, Anastasia Langanz, Leonie Marie Weskamm, Nele Wichern, Annika Bea, Johannes Brandi, Helena Fehling, Nils Groth, Melanie Lütkemeyer, Matthias Knödler, Henrik Nausch, Eirini Stivachti, Barbara Honecker, Hanna Lotter

**Affiliations:** 1 Research Group Molecular Infection Immunology, Bernhard Nocht Institute for Tropical Medicine, Hamburg, Germany; 2 Research Group Virus Immunology, Bernhard Nocht Institute for Tropical Medicine, Hamburg, Germany; 3 Institute for Infection Research and Vaccine Development (IIRVD), Center for Internal Medicine, University Medical Center Hamburg-Eppendorf, Hamburg, Germany; 4 Department for Clinical Immunology of Infectious Diseases, Bernhard Nocht Institute for Tropical Medicine, Hamburg, Germany; 5 German Center for Infection Research, partner site Hamburg-Lübeck-Borstel-Riems, Hamburg, Germany; 6 Fraunhofer Institute for Molecular Biology and Applied Ecology IME, Fraunhofer-Gesellschaft zur Förderung der angewandten Forschung e. V, Aachen, Germany; 7 Institute for Molecular Biotechnology, RWTH Aachen University, Aachen, Germany

**Keywords:** Sex-specific differences in vaccine response, CD64 (FcγRI), Classical monocytes, Mo-DCs, Classical antigen presentation, Cross-presentation, Intramuscular immunization, FcγR-mediated immune response

## Abstract

Sex-specific differences influence vaccine-induced immunity, with females generally mounting more robust immune responses than males. While enhanced antibody production in females is well established, the cellular mechanisms underlying these differences remain elusive. Monocytes and monocyte-derived dendritic cells contribute to vaccine-induced immunity by capturing antibody-antigen immune complexes via Fc *gamma* receptors (FcγRs), positioning them as potential mediators of sex-biased vaccine responses. Here, we investigated sex-specific differences in FcγRI (CD64)-expressing monocytic cells in humans and mice. Using flow cytometry, we demonstrate that healthy women display higher frequencies of CD64-positive monocytes and elevated CD64 expression in peripheral blood compared with men. In mice, a similar sex-specific pattern was consistently reproduced across diverse experimental settings, including in vitro and in vivo conditions. Functional in vitro assays revealed that classical monocytes from female mice mediated stronger classical antigen presentation to CD4⁺ T cells than male-derived monocytes in a CD64-dependent manner, as reflected by increased IFNγ production. In parallel, female-derived monocytes also displayed enhanced cross-presentation to CD8⁺ T cells; however, this effect occurred independently of CD64. Following intramuscular vaccination, flow cytometric analysis demonstrated that female mice exhibited increased frequencies of CD64-positive monocytes at the injection site, accompanied by higher CD64 surface expression levels on these cells. In addition, females showed significantly greater accumulation of monocytic cells in dorsal lymph nodes compared with males. Notably, castration of male mice enhanced monocytic recruitment to muscle tissue following immunization, indicating a role for sex hormones in regulating monocytic responses to vaccination. Together, these findings identify sex-specific regulation of CD64⁺ monocytic cells as a mechanism associated with enhanced antigen presentation in females and suggest that this pathway contributes to stronger vaccine-induced immune responses. In this context, our data underscore the relevance of biological sex in shaping Fcγ receptor-mediated immune mechanisms relevant to vaccination.

**Supplementary Information:**

The online version contains supplementary material available at 10.1186/s13293-026-00897-7.

## Highlights

Increased CD64⁺ monocyte frequencies and elevated CD64 expression in women, with a conserved sex-specific pattern in mice across in vitro and in vivo conditions.

Female-derived monocytes show enhanced classical antigen presentation to CD4⁺ T cells in a CD64-dependent manner.

Cross-presentation to CD8⁺ T cells by female-derived monocytes is also enhanced but occurs independently of CD64.

Following intramuscular immunization, female mice display increased proportions of CD64⁺ monocytes in muscle tissue and greater accumulation of monocytic cells in dorsal lymph nodes.

Castration of male mice increases monocytic recruitment to muscle tissue after immunization, implicating sex hormones in the regulation of monocytic responses.

These findings identify sex-specific regulation of CD64⁺ monocytes as a cellular mechanism contributing to sex-biased vaccine-induced immunity.

**Supplementary Information:**

The online version contains supplementary material available at 10.1186/s13293-026-00897-7.

## Abstract (plain language summary)

Vaccines often induce stronger immune responses in females than in males, with females producing higher antibody levels and achieving better protection after immunization. While these sex differences in antibody responses are well documented, less is known about differences in cellular immunity, particularly innate immune cells. Monocytes and monocyte-derived dendritic cells are key initiators of adaptive immune responses, as they capture antigens and present them to T and B cells. These cells express Fc gamma receptor I (CD64), which binds antibody-antigen immune complexes and promotes phagocytosis and antigen presentation, thereby linking innate and adaptive immunity. In this study, we show that healthy women exhibit higher frequencies of CD64-positive monocytes, and that these cells show higher levels of CD64 on their surface compared with men. A similar sex-specific pattern was observed in mice under different experimental conditions. After intramuscular vaccination, female mice showed increased accumulation of CD64-positive monocytes at the injection site and higher numbers of monocytic cells in the dorsal lymph nodes compared with males. In males, removal of testosterone enhanced monocytic cell recruitment to muscle tissue, indicating hormonal regulation of these responses. Functional analyses demonstrated that monocytes derived from females mediated stronger antigen presentation and activation of CD4⁺ T cells in a CD64-dependent manner. Moreover, female-derived monocytes also showed enhanced priming of CD8⁺ T cells, independent of CD64 function. Together, these findings suggest that higher numbers of CD64-positive monocytes in multiple tissues, together with CD64-dependent enhancement of classical antigen presentation, may contribute to stronger vaccine-induced immune responses in females.

**Supplementary Information:**

The online version contains supplementary material available at 10.1186/s13293-026-00897-7.

## Background

Sex-specific differences shape immune function, contributing to differential susceptibility to infectious and autoimmune diseases and to variation in vaccine efficacy and reactogenicity [1, 2]. These differences are driven by sex chromosomes and sex hormones, which together regulate immune cell development and activation [1, 2]. For example, women mount stronger humoral immune responses than men across inactivated, live-attenuated, and mRNA vaccines, often achieving antibody titers up to twofold higher than those of age-matched men [3, 4].

Although antibody titers remain key correlates of protection for most licensed vaccines [5], cellular immunity also plays a central role in vaccine-induced protection, particularly through antigen presentation and T cell priming. Evidence for sex-specific differences in antigen-presenting cell (APC) function is scarce, and the mechanisms underlying these differences remain poorly understood. Accordingly, defining the role of APCs is critical for understanding sex-specific vaccine responses. Among APCs, monocytes and dendritic cells (DCs) are particularly relevant in the vaccination context, as they efficiently capture antigen at peripheral sites and initiate T cell priming [6–8]. In humans, monocytes comprise classical CD14⁺CD16⁻, intermediate CD14⁺CD16⁺, and non-classical CD14⁻CD16⁺ subsets, whereas mice harbor classical Ly6C^hi^ and non-classical Ly6C^lo^ monocytes [9, 10]. Despite species-specific nomenclature, classical monocytes mediate cytokine production, phagocytosis, and antigen presentation, whereas non-classical subsets support tissue repair and vascular surveillance [10–12]. In both humans and mice, DCs are broadly classified into conventional DCs (cDCs), plasmacytoid DCs, and monocyte-derived DCs (Mo-DCs) [13]. cDCs represent developmentally distinct APCs specialized for antigen presentation, whereas Mo-DCs arise from monocytes under inflammatory conditions [11]. Monocytes and Mo-DCs, and to a lesser extent cDCs, express Fcγ receptors (FcγRs), enabling the recognition of IgG-opsonized antigens and initiation of inflammatory functions [13–16]. In both humans and mice, these processes are regulated by a diverse repertoire of activating and inhibitory FcγRs with distinct IgG subclass specificities, affinities, and signaling properties [14]. In humans, activating FcγRs include FcγRI (CD64), FcγRIIA/C (CD32A/C), and FcγRIIIA/B (CD16A/B), whereas mice express CD64, CD16, and CD16.2 (FcγRIV, the ortholog of human CD16A [17]). In both species, FcγRIIB (CD32B) serves as the sole inhibitory FcγR [14]. A productive immune response depends on the balanced integration of signals from activating and inhibitory FcγRs [18–20]. Following vaccination, pathogen-specific antibodies enable rapid opsonization upon antigen re-encounter, leading to immune complex (IC) formation and activation of Fc-mediated effector functions that contribute to protection [15]. Engagement of FcγRs by ICs enhances both MHCII-dependent classical antigen presentation to CD4⁺ T cells and MHCI-dependent cross-presentation to CD8⁺ T cells, positioning FcγRs as central regulators of antibody-dependent antigen presentation [15].

These pathways can be investigated ex vivo using ovalbumin (OVA)-based IC assays, which distinguish FcγR-dependent from FcγR-independent antigen presentation. In this system, IgG-opsonized OVA engages FcγRs on APCs, leading to FcγR-dependent internalization and processing, followed by activation of antigen-specific CD8⁺ and CD4⁺ T cells carrying transgenic T cell receptors (OT-I and OT-II) [18, 21, 22]. In contrast, direct loading of OVA or OVA-derived peptides bypasses FcγR engagement and isolates FcγR-independent antigen presentation, which is dependent on peptide availability and co-stimulatory signals, such as CD86-driven CD28 co-stimulation [23].

In mouse models, the high-affinity FcγR CD64 is commonly used to distinguish Mo-DCs from cDCs [24–27]. Following intramuscular (i. m.) immunization, Langlet et al. [24] demonstrated that CD64^+^ Mo-DCs and cDCs infiltrate the injection site and migrate to draining lymph nodes (LNs), where both populations function as APCs that prime T cells. Using ex vivo OVA-pulsing assays that bypass FcγR engagement, CD64⁺ Mo-DCs isolated from dorsal LNs primed CD8⁺ T cells more efficiently than cDCs while inducing comparable CD4⁺ T cell activation, indicating that CD64^+^ Mo-DCs possess strong antigen-presenting capacity [24]. Although the ability of CD64 to distinguish Mo-DCs varies between tissues and their identity remains debated because of overlap with cDCs and monocyte-derived macrophages, these findings nonetheless identify CD64⁺ monocytic cells as potent APCs [13, 28–30]. It remains unknown whether sex influences the abundance and function of these cells, modulates FcγR-mediated functions within this compartment, and contributes to sex-specific immune responses following vaccination.

Here, we showed that across humans and mice, females consistently exhibited higher frequencies of CD64⁺ classical monocytes accompanied by increased CD64 expression. Functionally, CD64-dependent classical antigen presentation resulted in enhanced CD4⁺ T cell activation by female-derived classical monocytes. In contrast, cross-presentation to CD8⁺ T cells by female-derived monocytes was enhanced but occurred independently of CD64. Following intramuscular Alum-OVA immunization, female mice exhibited increased frequencies of CD64⁺ monocytes at the injection site, along with enhanced accumulation of monocytic cells in the draining dorsal LNs. Castration in male mice increased monocytic cell recruitment to muscle tissue. Together, these findings identified sex-specific regulation of CD64⁺ classical monocytes as a plausible contributor to enhanced classical antigen presentation in females during i. m. vaccination.

## Materials and methods

### Human studies

All human studies were conducted in accordance with all relevant ethical regulations and approved by the ethics committee of the medical association of Hamburg (Ethik-Kommission der Ärztekammer Hamburg; permission number: PV5252). Blood samples were obtained from healthy donors of European ancestry (25–49 years of age, both sexes) under informed consent and anonymization.

### Isolation of human peripheral blood mononuclear cells

Peripheral blood mononuclear cells (PBMCs) were isolated from human donors by Biocoll (Biochrom) density gradient centrifugation. Whole blood was diluted 1:2 in DPBS, layered on Biocoll and centrifuged without brake (445 x *g*, 30 min, 21 °C). The PBMC layer was collected, washed twice with cold DPBS, and treated with erythrocyte lysis buffer for 5 min at room temperature. The lysis buffer consisted of 0.16 M NH_4_Cl and 0.1 M Tris (pH 7.6), prepared by mixing nine parts NH_4_Cl solution with one part Tris buffer. Lysis was stopped with cRPMI, followed by two additional washing steps.

### Flow cytometry analysis of human cells

**Fig. 1:**
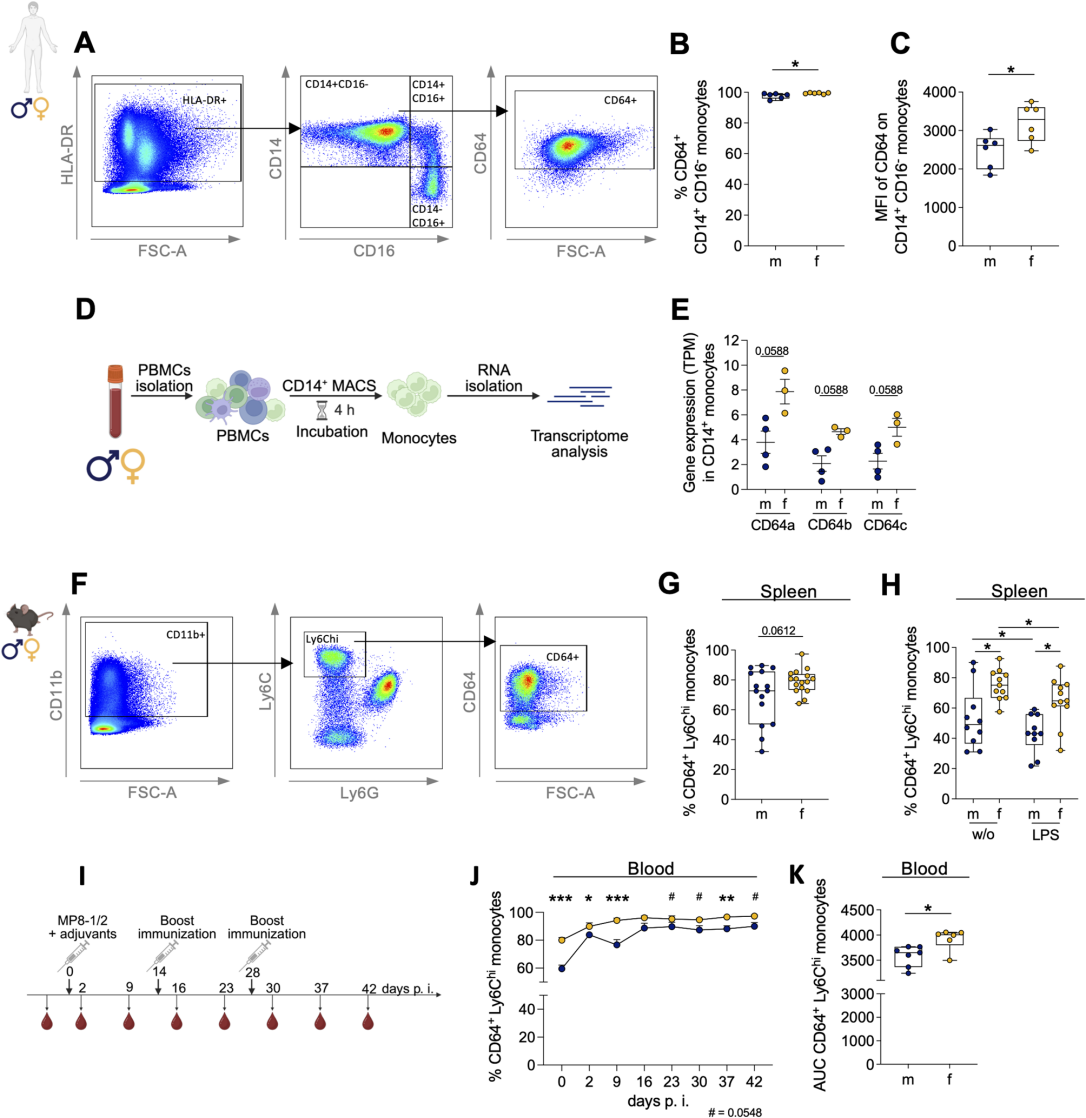
Sex-specific differences in CD64 expression on human and murine classical monocytes. (A) Gating strategy used to identify CD64⁺ classical monocytes (HLA-DR⁺CD14⁺CD16-) in fresh human peripheral blood. (B) Frequency and (C) median fluorescence intensity (MFI) of CD64⁺ monocytes in male and female donors. (D) Workflow for bulk RNA-seq of CD14⁺ monocytes from male and female donors. Created in BioRender. Lotter, H. (2026) https://BioRender.com/18ebt1l. (E) CD64 isoform transcript levels (transcripts per million, TPM) in CD14⁺ monocytes (n=3-4/sex). (F) Gating strategy used to assess CD64 expression on classical monocytes (CD11b⁺Ly6C^hi^Ly6G⁻) in murine blood and spleen. (G) Frequency of CD64⁺ Ly6Chi monocytes in the spleen of naive male and female mice (n=15-16/sex, 3 experiments) and (H) after 20 h *in vitro* LPS stimulation (n=10-11/sex, 2 experiments). (I) Schematic of intraperitoneal (i. p.) immunization with recombinant parasitic antigens and blood collection timeline. Created in BioRender. Lotter, H. (2026) https://BioRender.com/18ebt1l. (J) Frequency of CD64⁺ Ly6Chi monocytes in the blood during immunization (n=6-10/sex). (K) Area under the curve (AUC) analysis during immunization (n=6/sex). Statistical tests: Unpaired two-tailed t-tests, Mann-Whitney U tests or paired t-tests, with Holm-Šídák correction. Significance: *p < 0.05; **p < 0.01; ***p < 0.001.

For surface staining, 2 × 10^6^ cells per sample were washed with FACS buffer (DPBS + 1% charcoal-filtered FCS, to minimize influence of external hormones [31, 32]), stained with Zombie Aqua Fixable Viability dye (1:1000, BioLegend, according to the manufacturer’s protocol), washed twice, and incubated with fluorochrome-conjugated antibodies for 30 min at 4 °C in the dark (see Table 1). After two final washing steps, cells were fixed (DPBS + 4% PFA for 30 min at 4 °C in the dark), washed and resuspended in FACS buffer. Samples were measured using a BD^™^ LSRII and analyzed with Flow Jo^™^ software v10.8.1. The gating strategy is shown in Fig. 1A.

**Table 1:** Flow cytometry panel for phenotypic characterization of freshly isolated human PBMCs from whole blood

Antigen	Fluorochrome	Dilution	Clone	Company, Cat. No.
CCR2	APC	1:25	K036C2	BioLegend #357207
CCR5	AF488	1:25	J418F1	BioLegend #359103
CD14	AF700	1:25	M5E2	BioLegend #301822
CD16	PerCP	1:25	3G8	BioLegend #302030
CD64	APC-Fire750	1:25	10.1	BioLegend #305036
CX3CR1	PE-Cy7	1:25	2A9-1	BioLegend #341612
HLA-DR	BUV395	1:27	G46-6	BD #564040
Viability	Zombie Aqua	1:1000		BioLegend #423101

Measured on a BD^™^ LSRII flow cytometer

### RNA-sequencing and transcriptomic analysis

RNA-sequencing (RNA-seq) data were previously generated, analyzed and published in Sellau et al. [33]. In the present study, these data were reanalyzed to address a distinct biological question. Raw read counts were normalized to transcripts per million (TPM), enabling gene- and sample-level comparisons.

### Animal experiments

**Fig. 2:**
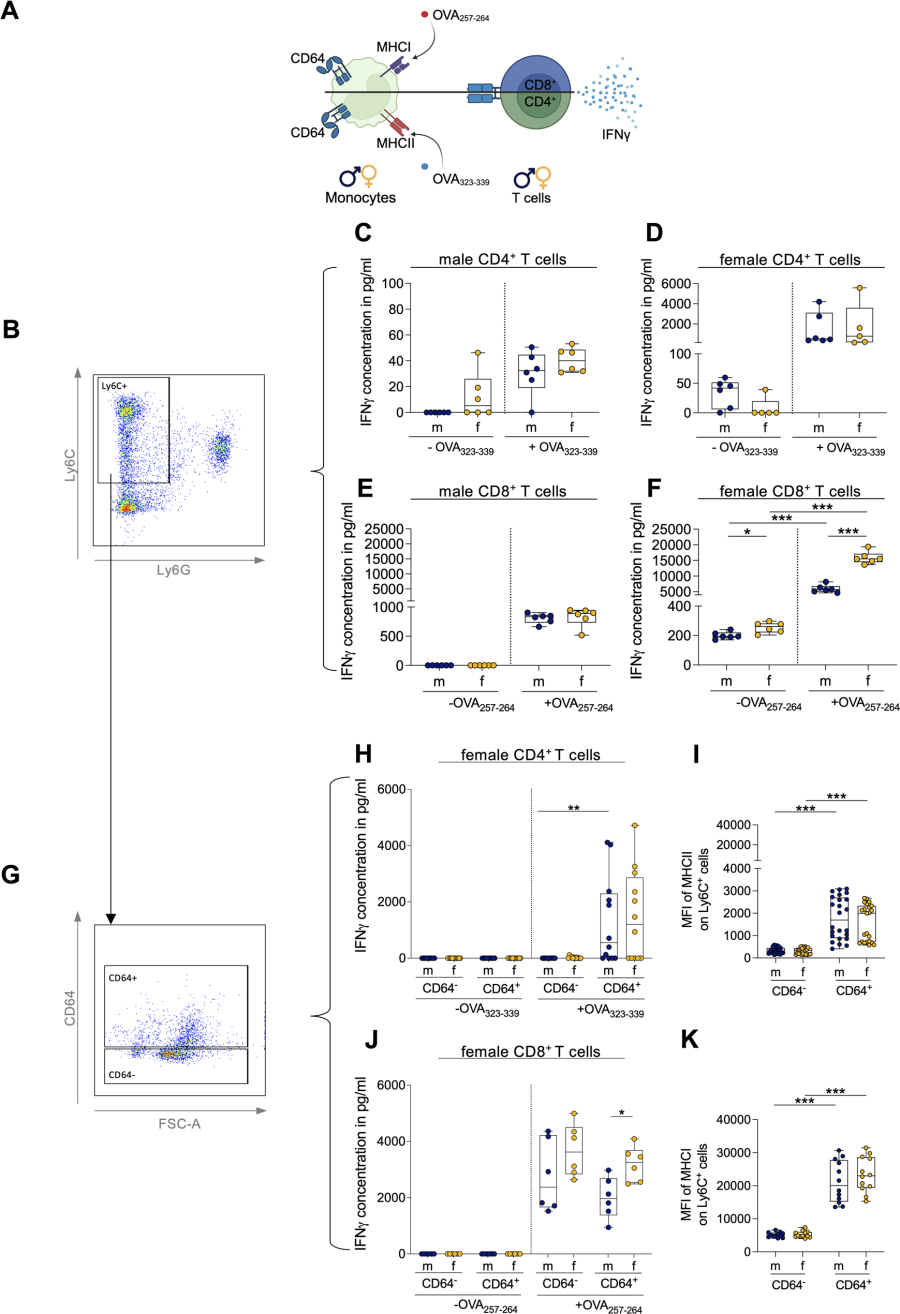
CD64 identifies a monocyte subset with enhanced classical T cell priming. (A) Schematic of classical and cross-T cell priming assay. Created in BioRender. Lotter, H. (2026) https://BioRender.com/3z3bm1x. (B, G) Gating strategy used to sort (B) Ly6C^+^ or (G) CD64^-^/CD64^+^ Ly6C^+^ monocytes from splenocytes of naive male and female mice. (C-D) Classical T cell priming by Ly6C⁺ monocytes, pulsed with OVA_323-339_ and co-cultured with CD4⁺ T cells from (C) male or (D) female OT-II mice for 72 h (n=5-6/sex). (E-F) Cross-T cell priming by Ly6C⁺ monocytes pulsed with OVA_257-264_ and co-cultured with CD8⁺ T cells from (E) male or (F) female OT-I mice for 24 h (n=6/sex). (H-J) CD64⁻ and CD64⁺ Ly6C⁺ monocytes from male and female mice, pulsed with OVA_323-339_ and co-cultured with female (H) OT-II CD4⁺ T cells or (J) OT-I CD8⁺ T cells for 24 h (n=6-12/sex; 1-2 experiments). (I) MHCII MFI on CD64⁻ and CD64⁺ Ly6C⁺ monocytes (n=23-24/sex; 4 experiments). (K) MHCI MFI on CD64⁻ and CD64⁺ Ly6C⁺ monocytes (n=12/sex; 2 experiments). IFNγ measured via ELISA. Unpaired two-tailed t-tests, Mann-Whitney U tests, paired t-tests or Wilcoxon tests, with Holm-Šídák correction. Significance: *p < 0.05; **p < 0.01; ***p < 0.001.

**Fig. 3:**
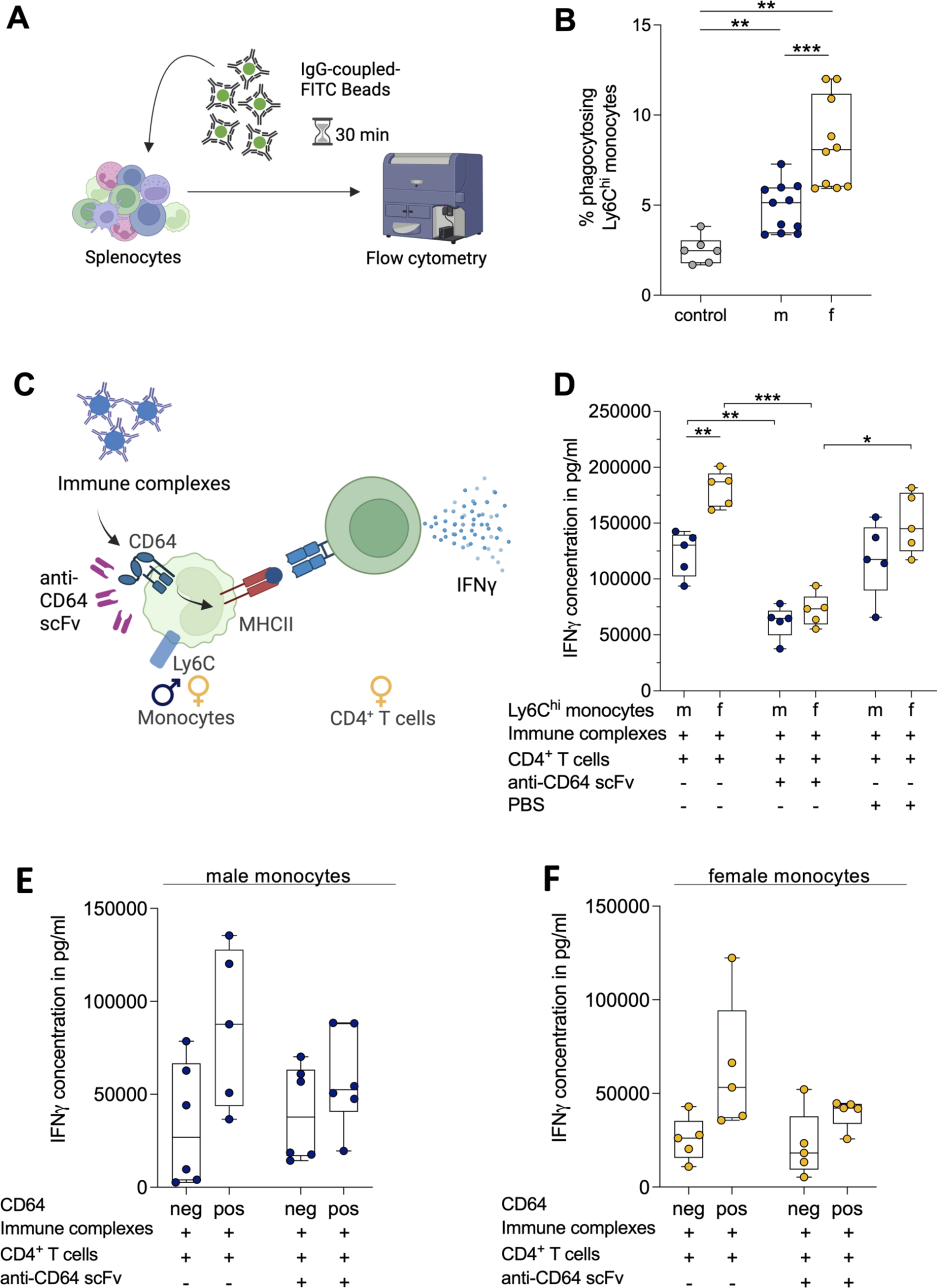
Female-derived monocytes exhibit enhanced FcγR-dependent phagocytosis and classical antigen presentation. (A) Schematic of phagocytosis assay. Created in BioRender. Lotter, H. (2026) https://BioRender.com/3z3bm1x. (B) Phagocytic efficiency of Ly6C^hi^ monocytes from male and female mice (n=10/sex, 2 experiments). Negative control: 4 °C condition from female mice (n = 6, 2 experiments). (C) Illustration for classical antigen presentation assay using immune complexes. Created in BioRender. Lotter, H. (2026) https://BioRender.com/3z3bm1x. (D) Antigen presentation by Ly6C^hi^ monocytes from male and female mice, incubated with OVA + anti-OVA IgG immune complexes (OVA-ICs) and co-cultured with CD4^+^ OT-II T cells (n=5/sex) for 72 h. (E-F) CD64^-^ and CD64⁺ Ly6C⁺ monocytes from (E) male or (F) female mice, treated with anti-CD64 antibody (H22), incubated with OVA-ICs and co-cultured with CD4^+^ OT-II T cells for 72 h (n=5-6/group, 1-2 experiments). IFNγ concentrations in culture supernatants were measured by ELISA. Statistical tests: unpaired two-tailed t-test, Mann-Whitney U test, paired t-test or paired Wilcoxon test, with Holm-Šídák correction. Significance: *p < 0.05; **p < 0.01; ***p < 0.001.

All murine studies were approved by federal health authorities of the state of Hamburg (permits: A008/2020, N041/2020; N001/2025 for in vivo experiments; T-008 and T-011 for organ removal) and conducted in accordance with institutional and local legislation. C57BL/6J and Thy1.1xOT-IxC57BL/6J mice were bred in the animal facility of the Bernhard Nocht Institute for Tropical Medicine (BNITM) or purchased from Charles River (Sulzfeld, Germany). C57BL/6J OT-II mice were obtained from the University Medical Center Hamburg-Eppendorf (UKE, Fig. 2H) or Charles River (Figs. 2C-D and 3D-F). All mice were housed under specific pathogen-free conditions and kept in individually ventilated cages (12 h day/night cycle, 50–60% humidity, 21 °C). Wild-type mice (8–19 weeks old) and OT-I/OT-II (9–17 weeks old) were euthanized by CO_2_ overdose, followed by cervical dislocation or cardiac puncture.

### Immunizations

For intraperitoneal (i. p.) immunization, recombinant parasitic metallopeptidases MP8-1 and MP8-2 from *Entamoeba histolytica* (*E. histolytica*) were used. The respective coding sequences were cloned into the pRSET expression vector and expressed in *Escherichia coli* (*E. coli*) shuffle cells. Recombinant proteins were purified via His-tag affinity chromatography and emulsified 1:1 in Freund’s adjuvant (final volume 150 µL). Primary immunization was performed using complete Freund’s adjuvant (Sigma-Aldrich); first and second booster immunizations were performed using incomplete Freund’s adjuvant (Sigma-Aldrich). Depending on the construct, mice received total antigen doses of 205 µg MP8-1 or 73 µg MP8-2 per mouse over a 4-week immunization period. Immunization with MP8-1 and MP8-2 induced comparable immune responses across all measured parameters. Therefore, data from both immunizations were combined for downstream analyses. Blood was collected via submandibular bleeding on days 2 and 9 after the primary and booster immunizations.

As an adjuvanted model vaccine, 5 µg OVA (Endofit™ InvivoGen) was mixed with Imject^®^ Alum (500 µg aluminum- and 500 µg magnesium hydroxide, Thermo Fisher Scientific), and 0.9% NaCl. Control mice received Alum in NaCl or NaCl alone. In a separate experiment, the licensed inactivated influenza split vaccine Influsplit Tetra 2021/2022 (2.4 µg hemagglutinin) was administered without adjuvant, with NaCl serving as control. Mice were injected i. m. with 20 µL per site into the *tibialis posterior* muscle bilaterally using a BD Micro-Fine U-100 insulin syringe (29G, 12.7 mm), adapted from Langlet et al. [24]. Muscle tissue and LNs were collected 6 days post immunization (p. i.).

### Gonadectomy of male and female mice

Wild-type male and female mice (8 weeks old) underwent bilateral gonadectomy via testicular or ovarian ligation, as described previously [34], under ketamine/medetomidine anesthesia. Postoperative analgesia included carprofen (5 µg/g bodyweight; 24 h after surgery) and meloxicam (1 µg/g bodyweight) in drinking water for up to 7 days post-surgery.

### Implantation of flutamide pellets

Wild-type male mice (11–19 weeks old) underwent subcutaneous implantation of flutamide or placebo pellets (100 mg/pellet, 60-day release; SA-152 or SC-111; Innovative Research of America) under isoflurane inhalation anesthesia. A small skin incision was made in the dorsal cervical region, and a subcutaneous pocket was created lateral to the vertebral column using blunt dissection. The pellet was inserted using forceps, and the incision was closed with two interrupted sutures. Postoperative analgesia was administered as described for gonadectomy.

### Isolation of murine immune cells

Spleens were dissociated through 70 μm cell strainers and washed with DPBS. Red blood cells were lysed using RBC lysis buffer according to the manufacturer’s instructions (RBC Lysis Buffer 10 X, BioLegend), followed by DPBS washes. Whole blood (EDTA tubes) was diluted in DPBS, and lysed similarly to spleen cells. LNs were mechanically dissociated by mashing through a 70 μm cell strainer and washed with DPBS. Muscle cells were isolated as described in [35, 36] with adaptations: Muscles were minced and digested in DMEM with collagenase type II (2 mg/mL, Merck) and dispase (0.5 mg/mL, Sigma-Aldrich) for 20 min at 37 °C. Following enzymatic digestion, muscle clumps were further dissociated by gentle pipetting, incubated for another 10 min in digestion medium, washed twice and filtered through a 70 μm cell strainer pre-wetted with 2 mL FCS.

### Isolation of spleen-derived CD4^+^ and CD8^+^ T cells

CD8⁺ or CD4⁺ T cells were isolated from OT-I or OT-II-derived splenocytes, respectively, using isolation kits according to manufacturer’s instructions (CD4+/CD8 + T cell Isolation Kit, mouse; Miltenyi). Purity was assessed by flow cytometry (Table 2).

**Table 2:** Flow cytometry panel for assessment of MACS-purified CD8^+^ or CD4^+^ T cell purity

Antigen	Fluorochrome	Dilution	Clone	Company, Cat. No.
CD19	AF488	1:200	6D5	BioLegend #115521
CD4	APC	1:200	RM4-5	BioLegend #100515
CD8a	PE	1:200	53 − 6.7	BioLegend #100707

Measured on a Cytek^®^ Aurora 5-laser-spectral flow cytometer

### Sorting of spleen-derived monocyte subsets using flow cytometry

Single cell suspensions were stained with fluorochrome-conjugated antibodies and incubated for 30 min at 4 °C in the dark (Tables 3 and 4). For MHCI staining, cells were pre-incubated with TruStain FcX™ (BioLegend, anti-mouse CD16/32, 5 µL/spleen, 15 min, 4 °C). Cells were filtered (30 μm) and sorted using a BD FACS ARIA™ III. Gating included debris and doublets exclusion and selection of CD11b^+^ monocytes (Fig S2 A).

**Table 3:** Flow cytometry panel for FACS sorting of monocytic cell populations for in vitro co-culture assays with CD8⁺ OT-I T cells

Antigen	Fluorochrome	Dilution	Clone	Company, Cat. No.
CD11b	APC-Cy7	1:16.67	M1/70	BioLegend #101225
CD64	FITC	1:16.67	#027	Sino Biological #50086E027
H-2K^b^ (MHCI)	PerCP-Cy5.5	1:33.3	AF6-88.5	BioLegend #116515
Ly6C	PE	1:33.3	HK1.4	BioLegend #128008
Ly6G	APC	1:25	1A8	BioLegend #127614

Sorted on BD FACS ARIA™ III cell sorter

**Table 4:** Flow cytometry panel for FACS sorting of monocytic cell populations for in vitro co-culture assays with CD4⁺ OT-II T cells

Antigen	Fluorochrome	Dilution	Clone	Company, Cat. No.
CD11b	PE-Dazzle594	1:16.67	M1/70	BioLegend #101256
CD64	FITC	1:16.67	#027	Sino Biological #50086-R027-F
I-A/I-E (MHCII)	PerCP-Cy5.5	1:33.3	M5/114.15.2	BioLegend #107626
Ly6C	PE	1:33.3	HK1.4	BioLegend #128008
Ly6G	APC	1:25	1A8	BioLegend #127614

Sorted on BD FACS ARIA™ III cell sorter

### Flow cytometry analysis of murine immune cells

Immune cells isolated from spleen, blood, muscle tissue and LNs were washed with DPBS and stained with Zombie UV™ viability dye (1:1000, BioLegend, 20 min, RT, dark), washed with FACS buffer, followed by surface staining with fluorochrome-conjugated antibodies for 30 min at 4 °C in the dark. Individual flow cytometry panels are shown in Tables 5, 6 and 7. Cells were washed with FACS buffer and fixed using the eBioScience™ Foxp3/Transcription Factor Staining Buffer Set (Thermo Fisher Scientific) for 30 min at 4 °C in the dark. Samples were measured using a BD™ LSRII, BD LSRFortessa™ or a Cytek^®^ Aurora 5-Laser-Spectral Flow Cytometer and analyzed with Flow Jo™ software v10.8.1. Gating strategy is shown in Fig S4 and S7.

**Table 5:** Flow cytometry panel for characterization of murine blood cells during MP8-1/2 immunization

Antigen	Fluorochrome	Dilution	Clone	Company, Catalog #
CD11b	BV510	1:400	M1/70	BioLegend #101263
CD16	APC	1:50	#002	Sino Biological #50,326-R002-A
CD16.2	PE-Cy7	1:100	9E9	BioLegend #149516
CD16/32	PerCP-Cy5.5	1:400	93	BioLegend #101324
CD64	FITC	1:200	#027	Sino Biological #50086-R027-F
Ly6C	PE	1:800	HK1.4	BioLegend #128008
Ly6G	BV785	1:700	1A8	BioLegend #127645
Viability	Zombie UV	1:1000		BioLegend #423107

Measured on a BD™ LSRII flow cytometer

**Table 6:** Flow cytometry panel for the characterization of muscle tissue and LNs following Alum-OVA immunization

Antigen	Fluorochrome	Dilution	Clone	Company, Cat. No.
CCR7	PE-Cy5	1:100	4B12	BioLegend #120114
CD11b	BV510	1:100	M1/70	BioLegend #101263
CD11c	BUV395	1:100	HL3	BD #564080
CD16	APC	1:50	#002	Sino Biological #50326-R002-A
CD16.2	PE-Cy7	1:200	9E9	BioLegend #149516
CD16/32	PerCP-Cy5.5	1:200	93	BioLegend #101324
CD19	BV750	1:400	6D5	BioLegend #115561
CD209α	PE	1:60	FAB8345P	R & D Systems #902404
CD24	APC-Fire750	1:300	M1/69	BioLegend #101840
CD3	PerCP	1:100	clone 145-2C11	BioLegend #100326
CD335 (NKp46)	AF647	1:100	29A1.4	BioLegend #137628
CD38	BV421	1:800	clone 90	BioLegend #102732
CD43	BUV496	1:200	S7	BD #741067
CD45	APC-Fire810	1:300	30-F11	BioLegend #103174
CD62L	BV711	1:800	clone MEL-14	BioLegend #104445
CD64	FITC	1:200	#027	Sino Biological #50086-R027
CD86	BV605	1:800	GL-1	BioLegend #105037
F4/80	BV650	1:100	BM8	BioLegend #123149
I-A/I-E (MHCII)	AF700	1:600	M5/114.15.2	BioLegend #107622
Ly6C	Pacific Blue	1:50	HK1.4	BioLegend #128014
Ly6G	BV785	1:400	1A8	BioLegend #127645
MERTK	PE-Dazzle594	1:100	2B10C42	BioLegend #151524
Viability	Zombie UV	1:1000		BioLegend #423107

Measured on a Cytek^®^ Aurora 5-laser-spectral flow cytometer

**Table 7:** Flow cytometry panel for phenotypic characterization CD64^−^ and CD64^+^ cells

Antigen	Fluorochrome	Dilution	Clone	Company, Cat. No.
CD11b	APC-Cy7	1:600	M1/70	BioLegend #101225
CD11c	PE-Fire700	1:50	QA18A72	BioLegend #161107
CD16	BV605	1:100	S17014E	BioLegend #158027
CD16.2	BV711	1:100	9E9	BioLegend #149529
CD172a	BV510	1:50	P84	BioLegend #144032
CD19	BV750	1:600	6D5	BioLegend #115561
CD209α	PE	1:60	FAB8345P	R & D Systems #902404
CD3	PE-Cy5	1:800	17A2	BioLegend #100273
CD32	PE-Cy7	1:200	S17012B	BioLegend #156409
CD335 (NKp46)	BV421	1:25	29A1.4	BD #562850
CD38	APC-Fire810	1:100	90	BioLegend #102745
CD4	PerCP	1:100	GK1.5	BioLegend #100431
CD45	Spark UV387	1:800	30-F11	BioLegend #103188
CD64	FITC	1:100	#027	Sino Biological #50086-R027F
CD8	BV570	1:100	53-6.7	BioLegend #100739
CD86	BV650	1:100	GL-1	BioLegend #105035
CD88	RB613	1:200	20/70	BD #758651
F4/80	BUV737	1:100	T45-2342	BD #749283
I-A/I-E (MHCII)	Spark-Red718	1:600	M5/114.15.2	BioLegend #107675
Ly6C	Pacific Blue	1:200	HK1.4	BioLegend #128014
Ly6G	BV785	1:200	1A8	BioLegend #127645
MERTK	PE-Dazzle594	1:100	2B10C42	BioLegend #151524
MHCI	RB545	1:200	M1/42	BD #756275
Viability	Live Dead Blue	1:1000		Invitrogen #L23105
XCR1	APC	1:100	QA20A05	BioLegend #109407

Measured on a Cytek^®^ Aurora 5-laser-spectral flow cytometer

### UMAP-based visualization for flow cytometry data

Spleen-derived lymphocytes and monocytes were gated to exclude dead cells, debris and doublets. CD11b^+^ Ly6C^+^ cells were subdivided into CD64⁻ and CD64^+^ cells. Uniform Manifold Approximation and Projection (UMAP; v4.0.4 [37]) was performed in Flow Jo™ software on 6 samples (54,000 events/sample, DownSample, v3.3.1), which were subsequently concatenated. UMAP dimensionality reduction was computed using the following markers CD11c, CD16, CD16.2, CD32, CD38, CD64, CD86, CD88, CD172a, CD209α, F4/80, MERTK, MHCI, MHCII and XCR1. Parameters: Euclidean distance, 15 nearest neighbors, a minimum distance of 0.5 and two components. UMAPs were visualized using R script Spectre [38].

### Phagocytosis assay

FcγR-mediated phagocytosis was assessed using the Phagocytosis Assay IgG FITC Kit (Cayman Chemical) following the manufacturer’s instructions. Splenocytes (2 × 10^6^ cells/well) were incubated with IgG-coated FITC-beads (1:100 in pre-warmed cRPMI, 30 min, 37 °C–4 °C). Based on prior reports demonstrating enhanced phagocytic capacity of female-derived cells, control experiments were performed using female-derived cells [39–41]. Following incubation, extracellular fluorescence was quenched using Trypan Blue. Cells were subsequently stained with Zombie UV™ viability dye (1:1000) and analyzed by flow cytometry (Table 8). Cells were washed in FACS buffer, and the FITC^+^ cells were acquired on BD LSRFortessa™ flow cytometer.

**Table 8:** Flow cytometry panel for characterization of phagocytosed FITC-coupled beads

Antigen	Fluorochrome	Dilution	Clone	Company, Cat. No.
Beads	FITC	1:100		Cayman Chemical #500290
CD11b	BV510	1:400	M1/70	BioLegend #101263
CD19	PE-Dazzle594	1:200	6D5	BioLegend #115554
CD86	AF700	1:100	GL-1	BioLegend #105024
F4/80	BV650	1:200	BM8	BioLegend #123149
Ly6C	PE	1:800	HK1.4	BioLegend #128008
Ly6G	BV785	1:800	1A8	BioLegend #127645
Viability	Zombie UV	1:1000		BioLegend #423107

Measured on a BD LSRFortessa™ flow cytometer

### Stimulation

Splenocytes from male and female mice were incubated with LPS (5 µg/mL; Sigma-Aldrich) for 20 h; control cells were incubated in cRPMI medium. Cells were subsequently stained with Zombie UV™ viability dye (1:1000) and the fluorochrome-conjugated antibodies (Table 9). Samples were acquired on BD LSRFortessa™ flow cytometer.

**Table 9:** Flow cytometry panel for characterization of murine splenocytes for in vitro analyses

Antigen	Fluorochrome	Dilution	Clone	Company, Cat. No.
CD11b	BV510	1:400	M1/70	BioLegend #101263
CD16	APC	1:50	#002	Sino Biological #50326-R002-A
CD16.2	PE-Cy7	1:100	9E9	BioLegend #149516
CD16/32	PerCP-Cy5.5	1:400	93	BioLegend #101324
CD19	PE-Dazzle594	1:200	6D5	BioLegend #115554
CD64	FITC	1:200	#027	Sino Biological #50086-R027F
CD86	AF700	1:100	GL-1	BioLegend #105024
F4/80	BV650	1:200	BM8	BioLegend #123149
Ly6C	PE	1:800	HK1.4	BioLegend #128008
Ly6G	BV785	1:800	1A8	BioLegend #127645
NKp46	AF647	1:200	29A1.4	BioLegend #137628
Viability	Zombie UV	1:1000		BioLegend #423107

Measured on a BD LSRFortessa™ flow cytometer

### Antigen presentation assay using OVA peptides

CD11b⁺Ly6C⁺ monocytes were FACS-sorted from spleens of naive male and female mice (FACSAria™ III) and depending on experimental setup, further separated based on CD64 expression into CD64⁻/⁺ subsets. For peptide-stimulation, 1 × 10^4^ (for OVA_257-264_) or 3 × 10^4^ (for OVA_323-334_) monocytes/well were seeded in 96-well plates in 100 µL cRPMI, centrifuged (445 x *g*, 5 min, 4°C), and incubated with 100 µL peptide (0.25 µg/mL in cRPMI) or RPMI. OVA_257-264_-pulsed cells were washed after 2 h and co-cultured with CD8⁺ OT-I T cells (5 × 10^4^ cells/well), while OVA_323-339_-pulsed cells received CD4⁺ OT-II T cells without prior washing (2 × 10^5^ cells/well). Co-cultures were incubated for 24–72 h at 37 °C and 5% CO_2_. Supernatants were stored at − 20 °C for subsequent IFNγ ELISA.

### Immune complex formation

ICs were prepared as described by Clarke et al. and Ellsworth et al. [42, 43] by incubating 300 µg anti-OVA antibody (Sigma-Aldrich), 15 µg OVA, and DPBS for 1 h at 37 °C and 5% CO_2_. ICs were pelleted (17,200 *g*, 30 min, 4 °C), resuspended in cold DPBS and quantified by BCA Assay (Thermo Fisher Scientific). ICs were stored at 4 °C and used within 48 h.

### Antigen presentation assay using immune complexes

Classical Ly6C^hi^ monocytes as well as Ly6C⁺ CD64⁻ and Ly6C⁺CD64⁺ cells were FACS-sorted from spleens of naive male and female mice. For selected conditions, cells from two mice of the same sex were pooled. In CD4⁺ T cell co-cultures, 1 × 10^5^ Ly6C^hi^ monocytes from each sex (Fig. 3D) or Ly6C^+^ CD64⁻/CD64⁺ monocytes from male mice (Fig. 3E) were seeded together with 2 × 10^5^ CD4⁺ T cells from OT-II mice per well, whereas 5 × 10^4^ CD64⁺ monocytes from female mice (Fig. 3F) were co-cultured with 1 × 10^5^ CD4⁺ T cells from OT-II mice. For CD8⁺ T cell assays, 1.5 × 10^5^ monocytes of both sexes (Fig. 3S) and 3 × 10^5^ CD8⁺ T cells from OT-I mice were used per well in all conditions.

Cells were centrifuged (445 x *g*, 5 min, 4 °C) after plating in 96-well plates. Where indicated, cells were incubated for 30 min at 4 °C with 100 µg/mL single chain variable fragment (scFv) H22, a CD64-specific Fc-free antibody fragment, hereafter referred to as anti-CD64 scFv [44, 45]. DPBS incubation for 30 min at 4 °C served as control. Afterwards, 10 µg/mL OVA-ICs (50 µL/well) were added and co-cultured with CD4⁺ OT-II T or CD8^+^ OT-I T cells. Samples were incubated 48–72 h (37 °C, 5% CO_2_). Supernatants were collected at 48 h (2 × 60 µL) and 72 h (1 × 60 µL) and stored at − 20 °C for IFNγ ELISA.

### IFNγ ELISA

Briefly, IFNγ concentrations in cell culture supernatants were quantified using the ELISA MAX™ Deluxe Set Mouse IFNγ (BioLegend) according to the manufacturer’s instructions.

### Semi-quantitative ELISA for antibody titer determination

OVA (1:40 in carbonate-bicarbonate buffer) was coated onto 96-well high-binding plates (Greiner Bio-One; 100 µL, overnight, 4 °C). After three washes, samples were blocked with 5% milk powder in PBS (5% MPBS, 100 µL, 1 h, RT) and washed. Serial dilutions of plasma from Alum- or Alum-OVA-immunized mice were added in 3% milk powder PBS (3% MPBS; 100 µL, 2 h, RT). After washing, samples were incubated with polyclonal rabbit anti-mouse Ig/HRP (1:2000 in 3% MPBS, 50 µL, 2 h, RT). After final washes, 100 µL substrate solution (BD OptEIA^™^ TMB Substrate Reagent Set, BD Biosciences) was added and the reaction was terminated after 25 min with 2 M H_2_SO_4_. Absorbance was read at 450 nm. Since antibody levels at day 6 p. i. were only modestly increased relative to pre-immune plasma and did not allow robust endpoint titer determination, antibody responses were quantified using the area under the curve analysis (AUC) of ELISA optical density (OD) values across the full serial plasma dilution series (1:10–1:20,480).

### Statistics

All statistical analyses were performed using GraphPad Prism v10.2.0. Normality was assessed using Shapiro-Wilk tests. For comparisons, unpaired or paired t-tests were used for normally distributed data, Mann-Whitney *U* or Wilcoxon tests otherwise. *P* values were adjusted using the Holm-Šídák method. Data are shown as box-and-whisker plots (median, IQR, min-max) with all individual values, as mean ± SEM (Fig. 1J) or as stacked bar plots depicting the mean cellular composition. Exact *p* values are shown in the graphs for comparisons with *p* < 0.1. Statistical significance levels were defined as follows: *p* < 0.05 (*),* p <* 0.01 (**), *p <* 0.001 (***), and *p* < 0.0001 (****).

## Results

### Sex-specific differences in CD64 expression on human and murine classical monocytes

Monocytes and Mo-DCs are central regulators of antigen presentation and T cell priming through FcγR-mediated pathways [6–8]. Despite well-established sex-specific differences in vaccine-induced immunity [3, 4], it remains unclear whether FcγR-dependent functions within this compartment are differentially regulated between males and females.

In our study, sex-specific differences in CD64 expression were first evaluated in fresh human peripheral blood monocytes from healthy reproductive-age donors using flow cytometry. Female donors exhibited a significantly higher frequency of classical HLA-DR⁺ CD14⁺ CD16⁻ CD64⁺ monocytes and increased CD64 MFI relative to male donors (Fig. 1A-C). Consistent with these protein-level differences, bulk RNA-seq of isolated CD14⁺ monocytes indicated higher transcript levels of all three CD64 isoforms in females; however, these differences did not reach statistical significance (Fig. 1D, E; *p* = 0.0588).

To determine whether this sex bias in CD64 expression is conserved across species, we next examined classical monocytes in naive C57BL/6J mice. Splenic CD11b^+^ Ly6C^hi^ classical monocytes showed a higher frequency of CD64^+^ cells in females; this difference was not statistically significant (*p* = 0.0612; Fig. 1F, G). After in vitro culture, the between-group difference was more pronounced under both basal (medium-only) and LPS-stimulated conditions, reaching statistical significance, with female-derived cells displaying a higher frequency of CD64⁺ Ly6C^hi^ monocytes (Fig. 1H). In contrast, CD32⁺ Ly6C^hi^ monocytes were more frequent in male-derived samples following in vitro incubation in medium alone (Fig S1, A, B). Upon LPS stimulation, their frequency decreased in both sexes, thereby abolishing the sex difference. This response contrasts with CD64, for which the sex difference persisted following LPS stimulation (Fig. 1H).

To test whether these sex-dependent differences persist during an in vivo immune response, we next applied an adjuvanted amoebic immunization approach, drawing on *E. histolytica* as a well-characterized system for studying sex-specific, monocyte-driven immune responses [33, 46]. Mice were immunized i. p. with two recombinant *E. histolytica* antigens in Freund’s adjuvant, followed by booster doses at weeks 2 and 4 (Fig. 1I). Flow cytometric analysis of serial blood samples revealed that female mice maintained significantly higher frequencies of CD64⁺ Ly6C^hi^ monocytes throughout the immunization course, consistent with the difference already present in the naive state (Fig. 1J). AUC analysis further confirmed the sustained elevation of CD64⁺ monocytes in females (Fig. 1K). In line with this sex-biased FcγR-profile, cells expressing the activating FcγR CD16.2 were similarly enriched in females, whereas cells expressing the inhibitory FcγR CD32 were significantly more abundant in males (Fig S1 C-F).

Taken together, analyses of human and murine samples under steady-state, in vitro, and in vivo conditions show that females consistently exhibit higher frequencies of CD64⁺ classical monocytes than males, indicating conserved sex-dependent CD64 expression.

### CD64 identifies a monocyte subset with enhanced classical T cell priming

Given that CD64 supports phagocytosis and antigen presentation, and that female individuals displayed higher frequencies of CD64⁺ classical monocytes, we next examined whether these differences translated into altered T cell priming. To first assess the FcγR-independent antigen presentation, Ly6C^+^ monocytes were pulsed with MHCII-restricted (OVA_323-339_) and MHCI-restricted (OVA_257-264_) peptides. Peptide-loaded monocytes were then co-cultured with OVA-specific CD4⁺ (OT-II) or CD8⁺ (OT-I) T cells. IFNγ production was used as a functional readout of productive T cell priming (Fig. 2A, B).

In classical T cell priming (OVA_323-339_ → CD4⁺ T cells), monocytes from both sexes induced comparable IFNγ responses, and no sex-dependent differences were observed. Female-derived OT-II T cells exhibited higher IFNγ production than male-derived OT-II T cells (Fig. 2C, D). In cross-T cell priming (OVA_257-264_ → CD8⁺ T cells), monocytes from male and female mice were equally effective when co-cultured with male-derived CD8⁺ OT-I T cells. Peptide pulsing increased IFNγ production in these co-cultures; however, this increase did not reach statistical significance (Fig. 2E). In contrast, when female-derived CD8⁺ OT-I T cells were used, peptide pulsing significantly enhanced IFNγ production. Moreover, female-derived monocytes induced significantly higher IFNγ production than male-derived monocytes, irrespective of peptide pulsing (Fig. 2F). This indicates a sex-specific enhancement in CD8⁺ T cell activation that becomes apparent only in the presence of female T cells. To focus on sex-specific differences induced by monocytes and exclude T cell-derived effects, all subsequent experiments were performed using female-derived OT-I and OT-II T cells.

To assess whether the expression of CD64 modulates T cell priming efficiency, Ly6C⁺ monocytes were sorted into CD64⁻ and CD64⁺ subsets (Fig. 2G). During classical antigen presentation, CD64⁺ monocytes from both sexes induced higher IFNγ production (Fig. 2H), reaching statistical significance in males but not in females. Consistently, MHCII expression was significantly elevated in CD64⁺ subsets, whereas no sex-dependent differences were observed in either MHCII expression or IFNγ production (Fig. 2H, I). Despite markedly increased MHCI expression on CD64⁺ monocytes, CD8⁺ T cell responses were comparable between CD64⁺ and CD64⁻ subsets (Fig. 2J, K). Instead, sex emerged as the primary determinant of cross-T cell priming efficiency: female-derived monocytes elicited significantly stronger CD8⁺ T cell responses than male-derived monocytes within the CD64⁺ subset, whereas responses were comparable in CD64⁻ cells; however, these differences did not reach statistical significance. Together, these findings indicate that the observed effect was independent of CD64 status (Fig. 2J, K).

More detailed phenotypic analysis of CD64⁻ and CD64⁺ Ly6C⁺ monocytes in an independent experiment showed that CD64⁺ Ly6C⁺ monocytes exhibited significantly higher frequencies of other FcγR⁺ populations (CD32⁺, CD16⁺, and CD16.2⁺), as well as significantly increased frequencies of CD86⁺ and CD209α⁺ (DC-SIGN) cells. These markers were partly co-expressed, indicating populations that may be highly effective in classical antigen presentation (Fig S2).

In FcγR-independent peptide presentation assays, CD4⁺ T cell priming was comparable between sexes, whereas CD8⁺ T cell responses were enhanced when female-derived monocytes were co-cultured with female-derived T cells. CD64⁺ monocytes exhibited enhanced classical CD4⁺ T cell priming, reaching statistical significance in males, which was consistent with their higher MHCII expression. In contrast, CD64 expression did not enhance cross-T cell priming, and sex remained the primary determinant of CD8⁺ T cell activation. These findings indicate that CD64 marks monocytes with enhanced classical T cell priming capacity, while sex-specific differences in cross-T cell priming occur independently of CD64. The contribution of FcγR-dependent pathways was therefore examined next.

### Female derived-monocytes exhibit enhanced FcγR-dependent phagocytosis and classical antigen presentation, with a key role for CD64

To assess whether FcγR-mediated functions differ between sexes, splenocytes were incubated in vitro with FITC-labeled, IgG-coupled beads and analyzed by flow cytometry (Fig. 3A). Within the CD11b⁺Ly6C^hi^ classical monocyte population, female mice displayed a significantly higher proportion of bead-internalizing cells than males, indicating enhanced FcγR-dependent phagocytic activity in females (Fig. 3B).

Since FcγR-mediated phagocytosis constitutes the initial step for IC-driven antigen processing, we next examined whether downstream FcγR-dependent antigen presentation also differs by sex. Ly6C^hi^ monocytes from male and female mice were incubated with OVA-ICs, together with CD4⁺ OT-II T cells (Fig. 3C). After 72 h, IFNγ levels were quantified. Female-derived monocytes elicited markedly higher IFNγ production by OT-II CD4^+^ T cells than male-derived monocytes, indicating superior classical antigen presentation capacity (Fig. 3D). To determine whether this sex difference depends on CD64, CD64 was blocked using a recombinant human anti-CD64 scFv, an Fc-free antibody fragment [44]. CD64 blockade significantly reduced overall IFNγ production and attenuated the sex difference. These findings suggest that CD64-mediated antigen uptake contributes to both the magnitude of the CD4⁺ T cell response and the observed female-biased enhancement in classical antigen presentation. In contrast, DPBS treatment reproduced IFNγ levels comparable to the untreated condition, although no statistically significant sex-dependent difference was detected (Fig. 3D).

To further validate the role of CD64 in antigen presentation, Ly6C⁺ monocytes from both sexes were sorted into CD64⁺ and CD64⁻ subsets (Fig. 2G). CD64⁺ monocytes demonstrated higher classical antigen presentation in both sexes, and this effect was attenuated by anti-CD64 treatment; however, none of the comparisons reached statistical significance (Fig. 3E-F).

Having demonstrated sex-specific differences and a CD64-dependent classical antigen presentation, we next examined whether similar sex- or CD64-associated differences extend to FcγR-mediated cross-presentation. In cross-presentation assays, no sex-specific differences were detected when Ly6C^hi^ monocytes were incubated with OVA-ICs and OT-I CD8⁺ T cells (Fig S3 A). In males, however, CD64⁺ monocytes were more effective than CD64⁻ monocytes in presenting OVA-ICs to CD8⁺ OT-I T cells; this difference did not reach statistical significance (Fig S3 B; *p* = 0.0897). This CD64-associated effect could not be assessed using anti-CD64 scFv, as anti-CD64 scFv itself triggered IFNγ production by CD8⁺ T cells even in the absence of monocytes or OVA-ICs (Fig S3 C).

This section examined how FcγR-mediated uptake contributed to the enhanced classical antigen presentation previously observed in CD64⁺ monocytes. Female-derived Ly6C^hi^ monocytes showed higher FcγR-dependent phagocytosis and stronger classical antigen presentation than male-derived monocytes, and CD64 blockade reduced IFNγ production and attenuated this female advantage.

### Intramuscular immunization induces sex-dependent CD64 expression on monocytic cells

**Fig. 4:**
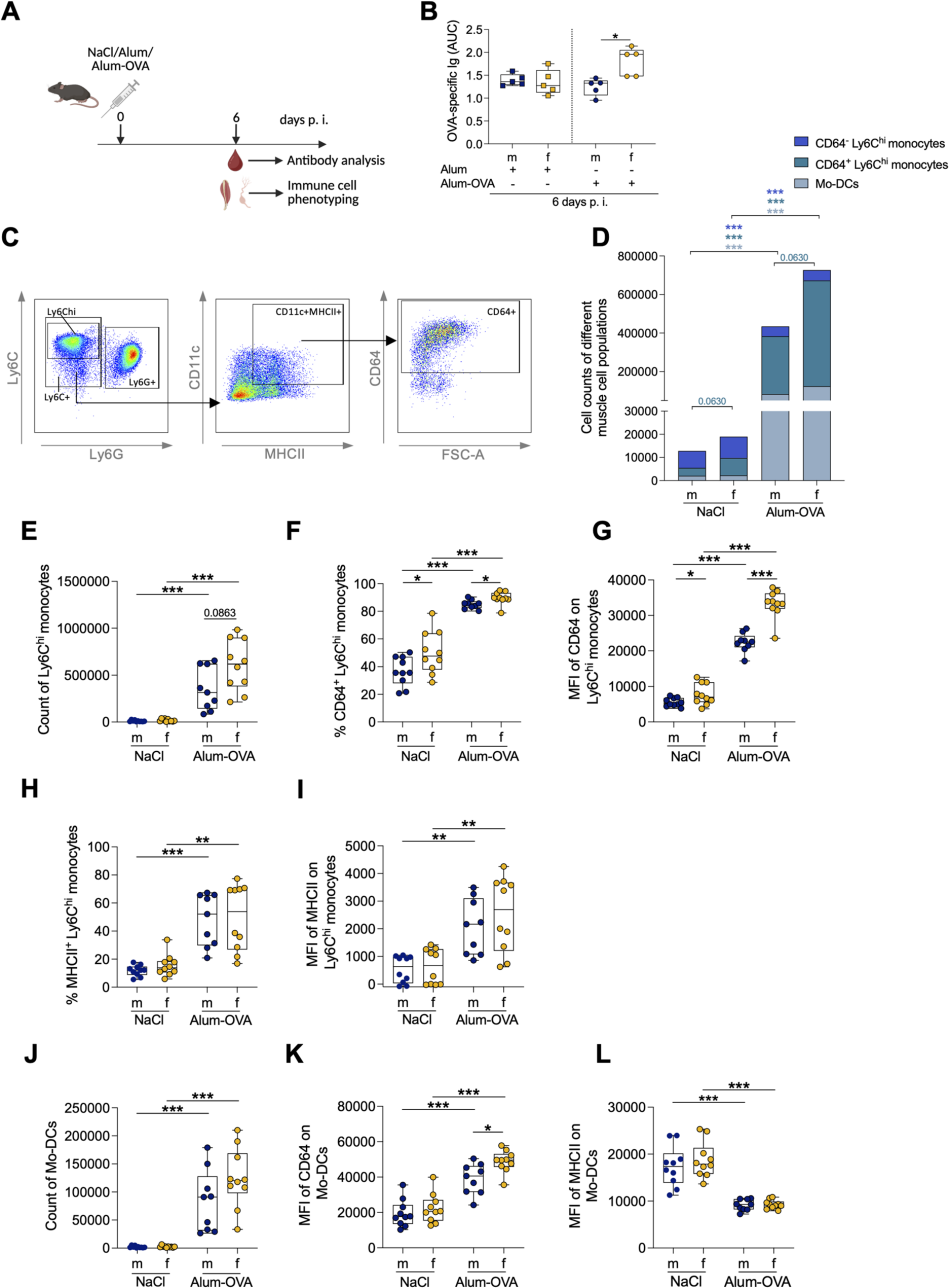
Intramuscular immunization induces sex-dependent CD64 expression on monocytic cells. (A) Schematic of experimental setup for Alum-OVA immunization. Created in BioRender. Lotter, H. (2026) https://BioRender.com/008celk. (B) OVA-specific immunoglobulin (Ig) titers 6 days p.i., measured by ELISA. AUC, Area under the curve. (C) Gating strategy used to identify Mo-DCs (CD11b^+^Ly6C^+^CD11c^+^MHCII^+^CD64^+^) in skeletal muscle, after exclusion of B cells (CD19), NK cells (NKp46) and macrophages (F4/80^hi^). (D) Monocytic cell populations in muscle tissue of male and female mice. (E) Counts of Ly6C^hi^ monocytes. (F) Frequency and (G) MFI of CD64^+^ Ly6C^hi^ monocytes. (H) Frequency and (I) MFI of MHCII^+^ Ly6C^hi^ monocytes. (J) Counts of Mo-DCs. (K) MFI of CD64 or (L) MHCII expression on Mo-DCs (n=9-10/sex, 2 experiments). Statistical tests: unpaired two-tailed t-test or a two-tailed Mann-Whitney U test with Holm-Šídák correction. Significance: *p < 0.05; **p < 0.01; ***p < 0.001.

Given that i. m. immunization represents the predominant route for vaccine delivery [24], we next assessed innate immune cell recruitment and monocytic phenotypes following i. m. immunization. Male and female mice were immunized with Alum-OVA, and immune cell composition in the injected muscle as well as plasma antibody responses were analyzed 6 days p. i. (Fig. 4A).

At this early post-immunization time point, antibody responses were already significantly higher in females than in males (Fig. 4B). Immunization-associated changes within monocytic populations at the site of antigen delivery were next evaluated using flow cytometry, as shown in Fig. 4C. The complete gating strategy is shown in Fig S4. Analysis of the *tibialis posterior* muscle revealed that females exhibited more CD64⁺ Ly6C^hi^ monocytes than males at baseline, and a similar pattern was observed following Alum-OVA immunization; however, these differences were not statistically significant (*p* = 0.0630). Immunization induced a significant increase in CD64⁻ and CD64⁺ Ly6C^hi^ monocytes and Ly6C⁺ CD11c⁺ MHCII⁺ CD64⁺ Mo-DCs in both sexes, together with recruitment of NK cells, neutrophils, and B cells (Fig. 4D, Fig S5 A). Although Ly6C^hi^ monocytes accumulated in both sexes, the increase was more pronounced in females, but the difference was not statistically significant (Fig. 4E, *p* = 0.0863).

In addition to changes in cell numbers, phenotypic activation of monocytic cells was examined. Females exhibited significantly higher frequencies and expression levels of CD64 on Ly6C^hi^ monocytes after NaCl and Alum-OVA immunization, with CD64 expression further increased after immunization (Fig. 4F-G). MHCII expression on Ly6C^hi^ monocytes increased significantly after immunization in both sexes, whereas CD86 was more strongly upregulated in females (Fig. 4H-I; Fig S5 E).

A similar pattern was observed among Mo-DCs. Their numbers increased after immunization in both sexes (Fig. 4J). Following immunization, CD64 expression on Mo-DCs was increased in both sexes and remained significantly higher in females (Fig. 4K), whereas MHCII expression decreased in both sexes (Fig. 4L). CD86 expression increased after immunization and was significantly higher in females (Fig S5 I).

Taken together, these data indicate that i. m. Alum-OVA immunization induces a more pro-inflammatory and activated monocytic environment in female-derived muscle tissue, characterized by elevated CD64 expression and increased co-stimulatory molecule expression across monocytic subsets.

### Sex-dependent recruitment of monocytic cells to dorsal lymph nodes following immunization

**Fig. 5:**
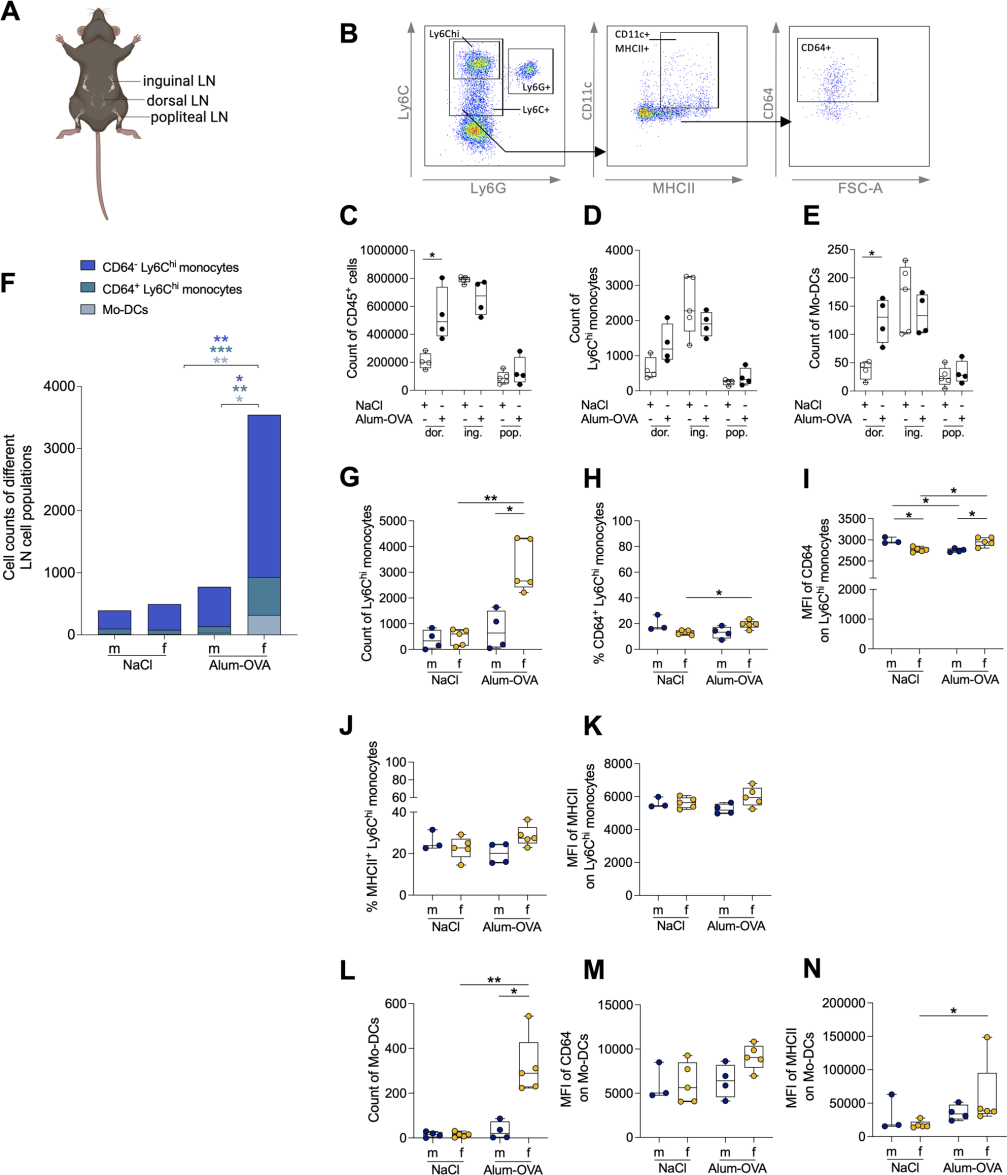
Sex-dependent recruitment of monocytic cells to dorsal lymph nodes following immunization. (A) Illustration of primary draining lymph nodes (LNs) following i. m. immunization in the *tibialis posterior* muscle. Created in BioRender. Lotter, H. (2026) https://BioRender.com/008celk. (B) Gating strategy used to identify Mo-DCs in LNs, after exclusion of B cells (CD19), NK cells (NKp46), and macrophages (F4/80^hi^). (C) Total CD45^+^ cell, (D) Ly6C^hi^ monocyte and (E) Mo-DC counts in female mice (n=4; dor.=dorsal; pop.=popliteal; ing.=inguinal). (F) Monocytic cell populations in dorsal LNs of male and female mice. (G) Count of Ly6C^hi^ monocytes. (H) Frequency and (I) MFI of CD64 expression on Ly6C^hi^ monocytes. (J) Frequency and (K) MFI of MHCII expression on Ly6C^hi^ monocytes. (L) Count of Mo-DCs. (M) MFI of CD64 expression (N) and MFI of MHCII expression on Mo-DCs (n=3-5/sex). One individual (male, NaCl) was excluded from the characterization of Mo-DCs and Ly6C^hi^ monocytes due to absence of these cell populations. Statistical tests: unpaired two-tailed t-test or a two-tailed Mann-Whitney U test with Holm-Šídák correction. Significance: *p < 0.05; **p < 0.01; ***p < 0.001.

To examine innate immune cell migration following Alum-OVA immunization, LNs previously defined as primary draining sites by Langlet et al. [24] were isolated from female mice 6 days p. i and analyzed by flow cytometry (Fig. 5A, B) [24]. The complete gating strategy is shown in Fig S6. Among the isolated LNs, the dorsal (iliac) LNs showed a significant influx of leukocytes (CD45⁺ cells) and Mo-DCs (Fig. 5C-E). Therefore, the dorsal LNs were selected for subsequent analyses.

Against this background, the immune cell composition of the dorsal LNs was compared between sexes. Following immunization, female-derived LNs displayed a significant increase in CD64⁻ and CD64⁺ Ly6C^hi^ monocytes, as well as Mo-DCs. None of these populations expanded in male-derived dorsal LNs, revealing a clear sex-specific difference in these monocytic subsets (Fig. 5F, G, L). Consistent with this overall increase in leukocyte recruitment, NK cells, neutrophils, and T and B cells were also present at significantly higher numbers in female-derived LNs (Fig S7 A).

Phenotypic analysis further supported enhanced activation of monocytic cells in female-derived dorsal LNs. Within Ly6C^hi^ monocytes, both the frequency of CD64^+^ cells and CD64 MFI were significantly increased in females (Fig. 5H, I), and female-derived cells also exhibited a higher frequency of CD86^+^ cells (Fig S7 E), while MHCII expression remained comparable between sexes (Fig. 5J, K). Mo-DCs in female-derived LNs also showed slightly higher CD64, MHCII, and CD86 expression than those in males; however, these differences were not statistically significant (Fig. 5M, N; Fig S7 I). Notably, Mo-DCs are generally rare in LNs and are even less abundant under steady-state or NaCl-injected conditions [24], particularly in males, which limited their characterization in some male samples.

Collectively, these data showed that the dorsal LNs of female mice exhibit stronger recruitment and activation of monocytic cells after Alum-OVA immunization, reflecting a more pronounced pro-inflammatory phenotype than in males.

### Sex hormone effects on monocytic cells and CD64 expression

**Fig. 6:**
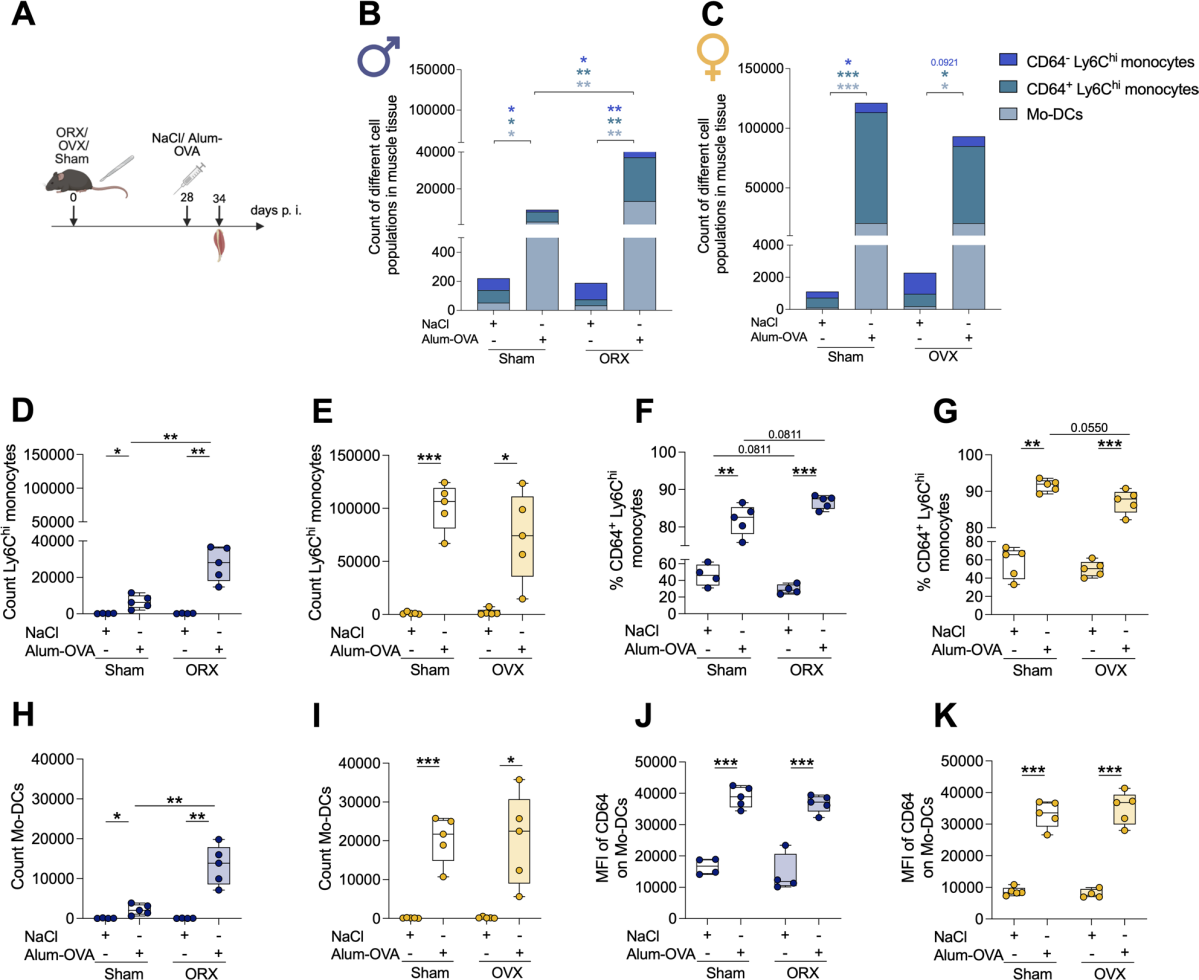
Sex hormone effects on monocytic cell infiltration and CD64 expression in immunized muscle. (A) Schematic of experimental setup of gonadectomy and i. m. Alum-OVA immunization. Created in BioRender. Lotter, H. (2026) https://BioRender.com/iufw2ar. (B-C) Monocytic cell populations in muscle tissue of sham-operated and gonadectomized (B) male and (C) female mice. (D-E) Ly6C^hi^ monocyte counts in muscle of (D) male and (E) female mice. (F-G) Frequency of CD64^+^ Ly6C^hi^ monocytes. (H-I) Mo-DC counts. (J-K) MFI of CD64 on Mo-DCs (n=4-5/group). Statistical analyses: unpaired two-tailed t-test or a two-tailed Mann-Whitney U test with Holm-Šídák correction. Significance: *p < 0.05; **p < 0.01; ***p < 0.001.

Given the pronounced sex-specific differences in monocytic cell recruitment and activation observed in muscle tissue and dorsal LNs, the contribution of gonadal sex hormones to these phenotypes was evaluated. Accordingly, male and female mice were surgically castrated (males=Orchiectomy, ORX; females=Ovariectomy, OVX) to ablate endogenous gonadal hormone production. After a four-week recovery period, mice received an i. m. Alum-OVA immunization, and muscle tissue and dorsal LNs were analyzed 6 days thereafter. Sham-operated mice served as controls (Fig. 6A).

In the muscle tissue of males, Alum-OVA immunization induced infiltration of monocytic cells (Fig. 6B). ORX markedly enhanced the recruitment of CD64⁻ and CD64⁺ Ly6C^hi^ monocytes and Mo-DCs, suggesting that androgens suppress the accumulation of immune cells in muscle at physiological hormone levels (Fig. 6B, D, H). This impact of gonadectomy extended to other immune cell populations: ORX led to a significant increase in neutrophils and NK cells p. i., whereas OVX did not alter monocytic or other immune cell numbers (Fig. 6C, E, I; Fig S8 A, B).

Consistent with the changes in cellular accumulation, analysis of cellular frequencies showed that immunization significantly increased the proportion of CD64⁺ Ly6C^hi^ monocytes in both intact and ORX males. The magnitude of increase was greater in ORX males, although this difference did not reach statistical significance (*p* = 0.0811; Fig. 6F). In females, immunization similarly increased CD64⁺ Ly6C^hi^ monocyte frequencies, with a reduced magnitude of increase following OVX; however, this difference was not statistically significant (*p* = 0.0550; Fig. 6G). Notably, these changes were not reflected in CD64 MFI (Fig S8 C, D), and CD64 expression on Mo-DCs remained unaffected (Fig. 6J, K).

To further dissect androgen-dependent mechanisms, we next pharmacologically blocked classical androgen receptor signaling using flutamide; however, flutamide treatment did not alter cell infiltration or the proportion of CD64⁺ Ly6C^hi^ monocytes compared with placebo controls (Fig S9). These data suggest that androgen-dependent effects on innate immune cell infiltration are not solely explained by classical androgen receptor signaling.

In contrast to muscle tissue, immune cell influx into the dorsal LNs of male mice remained limited and did not differ between intact and ORX groups (Fig S10 A, C, E). In females, both intact and OVX mice showed robust recruitment of monocytic cells after immunization (Fig S10 B, D, E). Notably, gonadectomy did not significantly affect the numbers of neutrophils, NK cells, B cells, or T cells in the dorsal LNs (Fig S10 M, N). Beyond changes in cell abundance, phenotypic analysis revealed subtle hormone-dependent effects on CD64 expression, with reduced CD64 levels on Ly6C^hi^ monocytes and Mo-DCs in OVX females (Fig S10 F, L).

Overall, these data indicate that androgens suppress innate immune cell infiltration into muscle tissue. In contrast, hormonal effects in the dorsal LNs were less pronounced. Together, these observations suggest that factors beyond circulating sex hormones, including genetic determinants, contribute to the observed sex-specific differences.

## Discussion

Sex-specific differences shape immune function across species, influencing infection outcomes, vaccine efficacy, and autoimmune disease risk [1, 2]. Although females often mount stronger humoral and cellular responses than males, the underlying cellular mechanisms remain poorly defined [2, 4, 47]. Because monocytic cells influence vaccine-induced immunity from early innate activation through antibody-dependent effector phases, defining sex-specific differences in these populations is essential to understand divergent vaccine responses. Here, we identify a conserved female-biased enrichment of CD64⁺ classical monocytes in humans and mice, accompanied by increased post-immunization accumulation of classical monocytes and Mo-DCs in female murine tissues. Functionally, CD64⁺ classical monocytes exhibit enhanced antigen-presenting capacity with potential implications for vaccine responsiveness.

Given that CD64 functions as an activating FcγR, FcγR-mediated pathways represent a plausible link between CD64 expression and enhanced antigen presentation. FcγR engagement enhances monocytic cell and DC function by promoting antigen internalization, phagocytosis, and both classical and cross-presentation [14, 19, 20, 48–51], and Fc-dependent mechanisms contribute substantially to protection in vaccination, infection, and monoclonal antibody-based therapies [52–60]. Within this receptor family, CD64 stands out as a high-affinity receptor that promotes IC-driven phagocytosis, cytokine production, cytotoxicity, and antigen presentation. CD64⁺ Mo-DCs and CD64⁺ cDCs internalize immune complexes more efficiently than their CD64⁻ counterparts, underscoring CD64 as a key mediator of these functions [28, 61]. Although mouse models for viral infections underscore context-dependent FcγR usage, with SARS-CoV-2 control relying primarily on CD16 and influenza immunity requiring both CD64 and CD16 [62, 63], these observations suggest that CD64 acts as a major contributor to FcγR-dependent mechanisms that influence vaccine-elicited immunity, albeit in a context-dependent manner.

Women exhibit higher frequencies of CD64⁺ classical monocytes and increased surface CD64 expression. Bulk RNA-seq analysis revealed a similar directional increase in FCGR1A transcript levels, although this did not reach statistical significance. Although sex-disaggregated expression differences are detectable across the *FCGR1* locus, functional consequences are expected to be limited to *FCGR1A*, given that *FCGR1B* and *FCGR1C* contain premature stop codons in the extracellular domain and are classified as pseudogenes or potentially soluble, non-membrane-anchored variants [64, 65]. Consistent with this interpretation, analysis of sex-disaggregated FCGR1A expression in the DICE database reveals modestly higher transcript abundance in females [66]. Quin et al. [67] similarly reported increased CD64 expression on female-derived monocytes from freshly isolated PBMCs by flow cytometry. Moreover, elevated CD64 expression in women aged 50–75 years with chronic low-grade inflammation provides further evidence for sex-biased regulation of CD64 [68]. Extending these observations, our study demonstrates that the female-biased enrichment of CD64⁺ classical monocytes is conserved across multiple experimental contexts in mice, including the naive state, LPS stimulation, and immunization with heterologous recombinant antigens.

Other FcγRs also exhibited sex-biased patterns, with females exhibiting higher expression of activating receptors (CD16, CD16.2) and males showing greater expression of the inhibitory receptor CD32. Among all receptors examined, CD64 displayed the most consistent and robust sex difference. Although CD64 is not exclusively expressed on Mo-DCs [13, 28], it is reliably present on these highly antigen-presenting-competent populations, underscoring its relevance for sex-specific differences in antigen processing.

To determine whether these patterns extended to physiologically relevant vaccine contexts, we examined the innate response after i. m. immunization. Following injection, female mice exhibited greater upregulation of CD64 on classical monocytes and Mo-DCs in muscle tissue, accompanied by increased accumulation of these cells in the dorsal LNs compared with males. In the literature, sex-specific differences in innate immune cell infiltration following i. m. vaccination, the most common route in humans [24], remain poorly defined. Studies focusing on downstream adaptive responses similarly report increased T follicular helper cells, germinal-center B cells, and plasmablasts in female LNs following i. m. pneumococcal conjugate vaccination [69], whereas a MIP-3α antigen-fusion DNA vaccine showed no detectable sex-specific differences in LN B or T cell numbers [70]. Together, these findings suggest that sex-biased recruitment and activation of immune populations after i. m. vaccination are context dependent, shaped by the nature of the antigen and adjuvant.

Gonadectomy experiments demonstrated that physiological levels of androgens suppress monocytic infiltration into muscle tissue. However, this effect is unlikely to represent a general inhibition of classical monocyte migration, as testosterone has been shown to promote Ly6C^hi^ monocyte infiltration in an infection, indicating a highly context-dependent regulation [33]. In our immunization model, androgen depletion modestly increased the frequency of CD64⁺ Ly6C^hi^ monocytes in male-derived muscle tissue p. i., whereas estrogen depletion in females led to a mild decrease in this population. In a separate human study conducted under different experimental conditions, using buffy coats and CD14-based MACS enrichment of monocytes prior to flow cytometric analysis, no sex-dependent differences in CD64 expression were observed in individuals of reproductive age. However, age-associated changes in the frequency of CD64⁺ monocytes emerged over time, with declining frequencies in women and increasing frequencies in men. These opposing findings are consistent with age- and likely hormone-associated influences on CD64 regulation [71]. Earlier studies categorizing FcγRs into CD64⁺ CD32⁺, and CD32^+^ subsets demonstrated that estrogens enhance, whereas androgens suppress, FcγR expression and function [72–74]. However, the magnitude of hormonal effects in our model was not significant, suggesting that additional mechanisms contribute to CD64 regulation. Recent analyses indicate that sex chromosome complement exerts extensive tissue- and cell-type-specific effects on autosomal gene expression [75, 76]. These findings raise the possibility that X- or Y-linked regulatory factors contribute to the regulation of FcγR expression, including CD64, which is encoded on chromosome 1 in humans (NCBI Gene 2209) and chromosome 3 in mice (NCBI Gene 14129), independently of hormonal signaling.

Functionally, female-derived monocytes displayed superior phagocytic capacity, consistent with long-standing observations of enhanced antigen uptake and processing in female-derived APCs [39–41, 70]. Classical and cross-presentation were assessed using two complementary approaches: an FcγR-independent assay, in which antigen presentation depends solely on MHC expression and co-stimulatory molecules, and a FcγR-dependent assay, in which FcγR-mediated uptake of ICs is required. In the FcγR-independent setting, classical antigen presentation did not differ between male- and female-derived monocytes. However, CD64⁺ monocytes were intrinsically more efficient APCs than CD64⁻ monocytes, even without CD64 engagement. This increased efficiency reflects their higher expression of MHCI, MHCII, CD16.2 and CD86, defining a more APC-competent baseline phenotype. In the FcγR-dependent assay, CD64⁺ monocytes from both sexes induced higher CD4⁺ T cell responses than CD64⁻ monocytes, and CD64 blockade reduced these responses. Although the subset comparisons did not reach statistical significance, the consistent directionality across experiments supports a functional contribution of CD64 to antigen presentation. Combined with the higher abundance of CD64⁺ classical monocytes in females, these findings suggest that enrichment of this subset may contribute to enhanced vaccine-induced protection in females. Indeed, Ly6C^hi^ monocytes from female mice were more efficient APCs than those from males. This convergence of phenotypic, functional and in vivo data strengthens the concept that sex-biased abundance of CD64⁺ monocytic cells provides females with an intrinsic advantage in antigen presentation. These observations parallel reports of superior antigen presentation by female-derived APCs [70, 77], and our data implicate CD64 as a potential mechanistic contributor to this sex difference.

In contrast to classical antigen presentation, cross-presentation followed a more complex pattern. In the FcγR-independent assay, female-derived monocytes exhibited enhanced cross-presentation irrespective of FcγR expression, indicating that sex-specific differences in this pathway are unlikely to be driven by FcγRs themselves. Instead, these differences are consistent with FcγR-independent factors, such as the availability of co-stimulatory molecules. In line with this interpretation, the co-stimulatory molecule CD86 was expressed at higher levels on monocytes from female mice. Notably, sex-specific differences became apparent only when monocytes were co-cultured with female-derived CD8⁺ T cells, suggesting that part of the observed bias originates from the T cell compartment. This observation is consistent with the notion that cross-presentation is shaped not only by the antigen-presenting capacity of APCs but also by the responsiveness of the interacting CD8⁺ T cells. Supporting this interpretation, previous studies have shown that female-derived CD8⁺ T cells exhibit intrinsically higher reactivity than their male-derived counterparts [78–80]. In parallel, FcγR-dependent cross-presentation was higher in male-derived CD64⁺ relative to CD64⁻ monocytes when co-cultured with female-derived CD8⁺ T cells; however, this difference was not statistically significant. Thus, while suggestive of enhanced antigen-presenting capacity, the data should be interpreted cautiously. Notably, this enhancement did not translate into detectable sex-specific differences within the Ly6C^hi^ monocyte compartment, indicating that CD64⁺ monocytes contribute only modestly to sex-specific variation in cross-presentation under these experimental conditions. Notably, sex-biased cross-presentation favoring females has been reported in DCs in other experimental settings, underscoring that the relative contribution of distinct cross-presenting cell types is highly context dependent [70].

Overall, our results identify CD64⁺ monocytic cells as sex-biased subsets that enhance classical antigen presentation in females. The conserved increase in CD64 expression and frequency across species, tissues, and immunization settings, together with the superior functional properties of CD64⁺ monocytes, suggest that this population may be an important determinant of sex-specific vaccine responses. To contextualize this role, it is important to consider the established contribution of monocytes to immunity. Monocytes are well established as drivers of vaccine-induced immunity: they rapidly produce IL-12, IL-1β, TNF, and IL-6 after immunization, serve as APCs during vaccine response, and their depletion delays CD8⁺ T cell priming [25, 81, 82]. Evidence from additional models supports a central role for CD64⁺ inflammatory monocytes in protective immunity. During vaccine-induced protection to *Helicobacter felis*, CCR2⁺ CD64⁺ monocytes accumulated at the infection site and were required for efficient pathogen clearance, as CCR2-deficient mice failed to recruit these cells and exhibited delayed protection [83]. Together, these observations reinforce a model in which CD64⁺ classical monocytes act as critical amplifiers of vaccine-induced immunity, and their female-biased enrichment offers a mechanistic basis for sex-specific differences in antigen presentation and vaccine responsiveness.

### Perspective and significance

Given the renewed interest in utilizing Fc-mediated immunity for universal influenza vaccines, antiviral antibody therapeutics, and tumor antigen vaccines [53, 84–86], understanding sex-specific regulation of FcγRs has become increasingly important. Beyond vaccination, the implications extend to autoimmunity. Approximately 80% of autoimmune disease patients are women [2, 87, 88], and elevated frequencies of CD64⁺ monocytes have been described across different autoimmune diseases. In humans, CD64 expression correlates with disease severity in rheumatoid arthritis [89] and systemic lupus erythematosus [90]. Supporting this notion, initial preclinical studies are exploring CD64 inhibition to disrupt IC-CD64 interactions as a strategy to dampen autoimmune responses [91]. In this context, the consistent enrichment of CD64⁺ monocytes in females may not only potentiate protective immune responses but also contribute to heightened susceptibility to autoimmune pathology.

### Conclusion

Taken together, our findings identify CD64⁺ Mo-DCs and Ly6C^hi^ classical monocytes as a sex-biased innate compartment, with the latter demonstrating enhanced antigen-presenting capacity, and position CD64 as a plausible mechanistic contributor to sex-specific immunity. Defining the extent to which CD64⁺ monocytes shape sex-specific differences in vaccine efficacy or autoimmune predisposition will require in vivo models that allow selective perturbation of this subset while avoiding compensatory FcγR signaling.

### Limitations of the study

Several limitations should be acknowledged. While our study reveals conserved sex-specific differences in CD64⁺ classical monocytes, the direct in vivo contribution of these cells to vaccine efficacy remains to be determined, as functional analyses were performed ex vivo. Future studies employing monocyte-specific genetic approaches or antibody-mediated CD64 blockade will be important to further validate these findings in vivo. In addition, our ex vivo assays were conducted using monocytes from naive mice, which may not fully reflect the functional properties of Mo-DCs following immunization. The low abundance of these populations, particularly in draining LNs, limited extensive functional characterization. Moreover, CD64 is not exclusively restricted to Mo-DCs and its discriminatory value varies across tissues, which should be considered when interpreting subset-specific effects. Additionally, human analyses were limited to ex vivo studies in healthy donors, providing a baseline framework for future investigations of vaccine-induced responses. Finally, sample sizes were limited in some experimental settings, particularly in the ORX, OVX, and flutamide treatment groups. Validation in larger cohorts would further increase statistical power and strengthen these observations.

## Electronic Supplementary Material

Supplementary Material 1

## Data Availability

The datasets generated and/or analyzed during the current study are available from the corresponding author upon reasonable request.

## Abbreviations

APCs: Antigen-presenting cells
AUC: Area under the curve
BCA: Bicinchoninic acid assay
cDCs: Conventional dendritic cells
DCs: Dendritic cells
*E. coli*: *Escherichia coli*
*E. histolytica*: *Entamoeba histolytica*
FcγR: Fc gamma receptors
Fig: Figure
i. m.: Intramuscular
i. p.: Intraperitoneal
IC: Immune complex
IFNγ: Interferon gamma
IgG: Immunoglobulin G
LNs: Lymph nodes
LPS: Lipopolysaccharide
Mo-DCs: Monocyte-derived dendritic cells
MP8: Metallopeptidase
MPBS: Milk-PBS
OD: Optical density
ORX: Orchiectomy
OT-I: OVA_257-264_-specific CD8+ T cells
OT-II: OVA_323-339_-specific CD4+ T cells
OVA: Ovalbumin
OVA_257-264_: Ovalbumin peptide (aa 257-264)
OVA_323-339_: Ovalbumin peptide (aa 323-339)
OVX: Ovariectomy
PBMCs: Peripheral blood mononuclear cells
RNA-seq: RNA sequencing
scFv: Single chain variable fragment
SEM: Standard error of the mean
Tab: Table
TPM: Transcripts per million
