# Supplementary material for: Sex shapes CD64 expression and vaccine-induced monocytic responses
Source: Biol Sex Differ. 2026 Apr 5;17:77. doi: 10.1186/s13293-026-00897-7 (PMC13081479; doi:10.1186/s13293-026-00897-7)
Supplement: Supplementary file 1 — Supplementary Material 1 [file 13293_2026_897_MOESM1_ESM.docx]

**Supplement
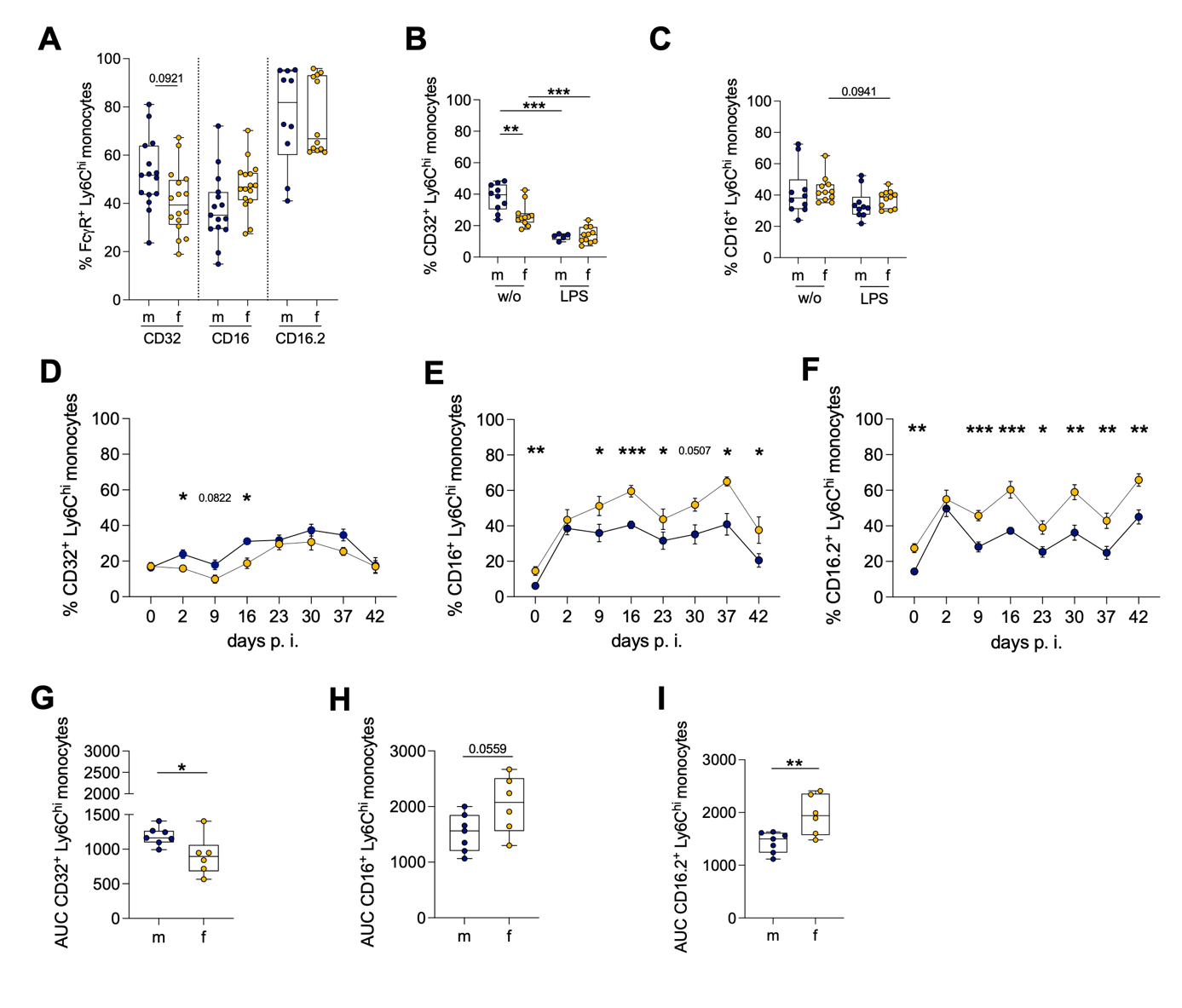
**

**Figure S1: Sex-specific expression of the Fc**γ**Rs CD32, CD16, and CD16.2.**

**(A)** Frequency of FcγR^+^ Ly6C^hi^ monocytes in the spleen of naive male and female mice (n=15-16/sex, 3 experiments) and **(B-C)** after 20 h of *in vitro* LPS stimulation (n=10-11/sex, 2 experiments). **(D-I)** Frequency and AUC analysis of (**D, G**) CD32^+^, (**E, H**) CD16^+^, (**F, I**) CD16.2^+^ Ly6C^hi^ monocytes in the blood during immunization with recombinant parasitic antigens (n=6-10 mice/sex). Statistical tests: Unpaired two-tailed *t*-tests, Mann-Whitney *U* tests, paired *t*-test, or paired Wilcoxon test with Holm-Šídák correction. Significance: **p* < 0.05; ***p* < 0.01; ****p* < 0.001.

**Figure S2: Characterization of spleen-derived CD64**⁻ **and CD64^+^ Ly6C^+^ monocytes by flow cytometry.**

**(A)** Gating strategy. **(B-H)** Surface marker expression on CD64⁻ and CD64^+^ Ly6C^+^ monocytes. Samples were downsampled and concatenated to 324,000 events (DownSample_v3.3.1). UMAPs were generated to visualize marker co-expression; representative male UMAPs are shown (UMAP v4.0.4: Euclidean distance, 15 nearest neighbors, minimum distance = 0.5, 2 components). UMAPs were visualized using the R script Spectre (38) (n=6/sex). Statistics: unpaired two-tailed *t*-test or a two-tailed Mann-Whitney *U* test with Holm-Šídák correction. Significance: **p* < 0.05; ***p* < 0.01; ******p* < 0.001.**

**Figure S3: Cross-presentation capacity of monocytic cells.**

**(A) Cross**-presentation by Ly6C^hi^ monocytes from male and female mice, incubated with OVA-ICs ± anti-CD64 scFv and co-cultured with CD8^+^ OT-I T cells for 48 h (n=5/sex). **(B)** CD64^-^ and CD64⁺ Ly6C⁺ monocytes from male mice, treated with anti-CD64 scFv, incubated with OVA-ICs and co-cultured with CD8^+^ OT-I T cells for 48 h (n=5/group). **(C)** CD8^+^ OT-I T cells incubated with OVA-ICs ± anti-CD64 scFv for 48 h (n=2). IFNγ was measured by ELISA. Statistical tests: unpaired two-tailed *t*-test or Mann-Whitney *U* test for sex comparisons; paired *t*-test or paired Wilcoxon test for treatment comparisons with Holm-Šídák correction. Significance: **p* < 0.05; ***p* < 0.01.

**Figure S4: Representative gating strategy for identification of immune cell subsets in muscle tissue.**

**Singlets and live CD45^+^ were gated, followed by exclusion of NKp46^+^ NK cells. CD19^+^ B cells were separated, while NKp46**⁻**CD19**⁻**F4/80^negtoint^CD11b^+^ cells were subdivided into Ly6G^+^ neutrophils, Ly6C^hi^ monocytes, and Ly6C^+^ cells. Ly6C^+^ cells were further analyzed for CD11c, MHCII, and CD64 expression.** Within the CD19⁻ NKp46⁻ compartment, CD11b⁻ CD3⁺ cells were defined as T cells.

**Figure S5: Innate and adaptive immune cell composition and Fc**γ**R/CD86 expression in Ly6C^hi^ monocytes and Mo-DCs from muscle tissue.**

**(A)** Cell counts of neutrophils, NK cells, B cells, and T cells in muscle tissue from naive male and female mice following Alum-OVA immunization, with NaCl-treated mice serving as controls (n=5-10/sex; 2 experiments). **(B-D)** Frequency of FcγR^+^ and **(E)** CD86^+^ Ly6C^hi^ monocytes and **(F-H)** frequency of FcγR^+^ and **(I)** CD86^+^ Mo-DCs in muscle tissue (n=9-10/sex, 2 experiments). Statistical tests: Unpaired two-tailed *t*-tests or Mann-Whitney *U* tests, with Holm-Šídák correction. Significance: **p* < 0.05; ***p* < 0.01; ******p* < 0.001.**

**Figure S6: Representative gating strategy for identification of immune cell subsets in dorsal LNs.**

**Singlets and live CD45^+^ were gated, followed by exclusion of CD19^+^ B cells and NKp46^+^ NK cells. Within the NKp46**⁻**CD19**⁻ **population, F4/80^negtoint^CD11b^+^ cells were subdivided into Ly6G^+^ neutrophils, Ly6C^hi^ monocytes, and Ly6C^+^ cells. Ly6C^+^ cells were further analyzed for CD11c, MHCII, and CD64 expression.** Within the CD19⁻ NKp46⁻ compartment, CD11b⁻ CD3⁺ cells were defined as T cells.

**Figure S7: Innate and adaptive immune cell composition and Fc**γ**R/CD86 expression in Ly6C^hi^ monocytes and Mo-DCs from dorsal LNs.**

**(A)** Cell counts of neutrophils, NK cells, B cells, and T cells in LNs of male and female mice following Alum-OVA immunization, with NaCl-treated mice serving as controls. **(B-D)** Frequency of FcγR^+^ and **(E)** CD86^+^ Ly6C^hi^ monocytes and **(F-H)** frequency of FcγR^+^ and **(I)** CD86^+^ Mo-DCs in the LNs (n=3-5/sex). Statistical tests: Unpaired two-tailed *t*-tests or Mann-Whitney *U* tests, with Holm-Šídák correction. Significance: **p* < 0.05; ***p* < 0.01.

**Figure S8: Innate and adaptive immune cell composition and CD64 MFI on Ly6C^hi^ monocytes in muscle tissue following gonadectomy and Alum-OVA immunization.**

**(A, B)** Immune cell counts in muscle tissue of **(A)** male and **(B)** female mice immunized four weeks after gonadectomy (ORX or OVX) (n=4-5/group). **(C-D)** **MFI of CD64 on Ly6C^hi^ monocytes** in muscle tissue of sham-operated and gonadectomized male **(C) and female (D) mice** (n=4-5/sex). Statistical tests: unpaired two-tailed *t*-tests or Mann-Whitney *U* tests, with Holm-Šídák correction. Significance: **p* < 0.05; ***p* < 0.01; ******p* < 0.001.**

**Figure S9: Effect of flutamide treatment on monocytic cell populations in muscle and LNs.**

**(A) Timeline of pellet implantation.** Created in BioRender. **(B, E)** Monocytic cell populations in **(B)** muscle and **(E)** LNs of placebo- and flutamide-treated male mice. Counts of Ly6C^hi^ monocytes and CD64^+^ Ly6C^hi^ monocyte frequencies **(C, D)** in muscle tissue and **(F, G)** LNs (n=3-5/group). One individual (male, placebo, NaCl) was excluded from the characterization of Ly6C^hi^ monocytes in the LNs due to absence of these cell populations. Statistical tests: unpaired two-tailed *t*-test or a two-tailed Mann-Whitney *U* test with Holm-Šídák correction. Significance: **p* < 0.05; ***p* < 0.01; ******p* < 0.001.**

**Figure S10: Immune cell composition and phenotypic characterization of monocytic cells in dorsal LNs following gonadectomy and immunization.**

**(A-B)** Monocytic cell populations in dorsal LNs of sham-operated and gonadectomized male **(A)** and female **(B)** mice. **(C-D)** Ly6C^hi^ monocyte counts. **(E-F)** Frequency of CD64^+^ Ly6C^hi^ monocytes. **(G-H)** MFI of CD64 on Ly6C^hi^ monocytes. **(I-J)** Counts of Mo-DCs. **(K-L)** MFI of CD64 on Mo-DCs (n=3-5/group). **(M-N)** Immune cell counts in dorsal LNs of gonadectomized **(M)** male and **(N)** female mice following immunization (n=4-5/group). Three male individuals (sham, NaCl) and one female individual (sham, NaCl) were excluded from the characterization of Mo-DCs due to the absence of these cell populations. Statistical analyses: unpaired two-tailed *t*-test or a two-tailed Mann-Whitney *U* test with Holm-Šídák correction. Significance: **p* < 0.05; ***p* < 0.01; ******p* < 0.001.**
